# Intricacies in arrangement of SNP haplotypes suggest “Great Admixture” that created modern humans

**DOI:** 10.1186/s12864-017-3776-5

**Published:** 2017-06-05

**Authors:** Rajib Dutta, Joseph Mainsah, Yuriy Yatskiv, Sharmistha Chakrabortty, Patrick Brennan, Basil Khuder, Shuhao Qiu, Larisa Fedorova, Alexei Fedorov

**Affiliations:** 1 Program in Bioinformatics and Proteomics/Genomics, University of Toledo, Health Science Campus, Toledo, 43614 OH USA; 2 Program in Biomedical Sciences, University of Toledo, Health Science Campus, Toledo, 43614 OH USA; 3 Department of Medicine, University of Toledo, Health Science Campus, Toledo, 43614 OH USA; 4 GEMA-biomics, Ottawa Hills, OH 43606 USA

**Keywords:** Population genetics, Computational biology, Bioinformatics, Inheritance, Genes

## Abstract

**Background:**

Inferring history from genomic sequences is challenging and problematic because chromosomes are mosaics of thousands of small Identicalby-descent (IBD) fragments, each of them having their own unique story. However, the main events in recent evolution might be deciphered from comparative analysis of numerous loci. A paradox of why humans, whose effective population size is only 10^4^, have nearly three million frequent SNPs is formulated and examined.

**Results:**

We studied 5398 loci evenly covering all human autosomes. Common haplotypes built from frequent SNPs that are present in people from various populations have been examined. We demonstrated highly non-random arrangement of alleles in common haplotypes. Abundance of mutually exclusive pairs of common haplotypes that have different alleles at every polymorphic position (so-called Yin/Yang haplotypes) was found in 56% of loci. A novel widely spread category of common haplotypes named Mosaic has been described. Mosaic consists of numerous pieces of Yin/Yang haplotypes and represents an ancestral stage of one of them. Scenarios of possible appearance of large number of frequent human SNPs and their habitual arrangement in Yin/Yang common haplotypes have been evaluated with an advanced genomic simulation algorithm.

**Conclusions:**

Computer modeling demonstrated that the observed arrangement of 2.9 million frequent SNPs could not originate from a sole stand-alone population. A “Great Admixture” event has been proposed that can explain peculiarities with frequent SNP distributions. This Great Admixture presumably occurred 100–300 thousand years ago between two ancestral populations that had been separated from each other about a million years ago. Our programs and algorithms can be applied to other species to perform evolutionary and comparative genomics.

**Electronic supplementary material:**

The online version of this article (doi:10.1186/s12864-017-3776-5) contains supplementary material, which is available to authorized users.

## Background

The origin of modern humans has long been a topic of debate and is still an area of active research. The discussion of human evolution has largely progressed around two key models namely the ‘out of Africa’ versus the ‘multi-regional’ models. While the most widely accepted ‘out of Africa’ hypothesis proposes that *Homo sapiens* evolved in Africa before migrating across the world [1–4], the opposing ‘multi-regional’ model proposes that intermingling of the various populations evolving in several regions over a long period of time resulted in the emergence of the modern *Homo sapiens* species [5, 6]. The events leading to the origin of *Homo sapiens* took place long ago, so our direct knowledge of human evolution is based on a limited number of fossils of archaic hominoid individuals discovered in different parts of the world. Researchers have widely used genomic molecular markers such as the mitochondrial DNA (mt-DNA) and the non-recombining region of Y chromosome (NRY) to study different aspects of human evolution. These markers are transmitted uniparentally (mt-DNA maternally and NRY paternally) and thus have their own limitations [7]. The recent advancement in next generation sequencing (NGS) has made large scale sequencing of human genomes affordable, and has led to a huge amount of genome wide sequencing data from large population cohorts. Modern human genomes preserve and carry signatures of many events in human evolution such as population bottlenecks, migration, admixture, natural selection and genetic drift, and therefore serve as reliably informative resources for elucidating the history of mankind. The 1000 Genomes database includes genetic information of 26 populations belonging to the African, Asian, European and American ancestry. This comprehensive resource on human genetic variation with diverse populations is ideal for the assessment of humans on a genomic scale. Recently our team computationally processed this database and demonstrated that very rare genetic variants (vrGVs, whose frequencies are less than 0.2%) are valuable markers for deciphering distant human relatedness [8, 9]. This examination brought to light the human migration routes and admixture that happened up to ten thousand years ago. However, to reveal more distant events in the history of mankind, genetic variants (GVs) with higher frequencies should be assessed. Keeping this in mind, here we investigated the distribution and structure of haplotypes built from the most frequent GVs (whose minor allele frequencies (MAF) are >25%) in people from Africa, America, Asia, and Europe (>90% of these GVs are SNPs). Surprisingly, intricacies of dynamics of frequent GVs and their dependence on selection, recombination, and population structure have been investigated in only several papers [10–15]. In this paper we have examined why modern humans have a strikingly large number (2.9 million) of frequent GVs. These frequent GVs were studied not individually but in haplotypes – groups of 50 adjacent and closely linked GVs. Such haplotypes were analyzed in 5398 segments along all autosomes. In a vast majority of cases a segment contains a few common haplotypes (CHs) that are widespread in 10%–90% of people from all continents. Below we focus our research specifically on CHs because they might have existed in populations for hundreds of thousands years and remain the same in a number of people from different populations. Thus, CHs may be of functional importance and their spread among populations and continents may reveal critical events occurred with ancient populations. Intriguingly, CHs very often exist in mutually exclusive pairs. The two individual haplotypes from such a mutually exclusive pair have different alleles practically at every GV site. Originally, this phenomenon was investigated by Zhang with co-authors and they named these mutually exclusive haplotype pairs as “Yin” and “Yang” haplotypes [16]. By analyzing common haplotypes in 62 random genomic loci and 85 gene-coding regions in humans, the Zhang *et al.* study proposed that the Yin/Yang haplotypes are abundant throughout the human genome and are genetic signatures that emerged prior to the African diaspora. Further, the peculiarities of Yin/Yang haplotype structures have been examined by Curits and Vine [17, 18]. Here we confirmed the widespread distribution of Yin/Yang haplotypes in humans and in addition revealed another widely distributed haplotype pattern, which we named “Mosaic”. The Mosaic haplotypes are built from multiple small pieces of Yin/Yang haplotypes.

To understand arrangement of alleles in common haplotypes, computer simulations of genome changes are very effective. Nowadays there are dozens of well-recognized computer programs capable of performing such investigations [19]. Here we specifically used whole-genome forward simulations with GEMA program [20, 21]. This algorithm is unique from others because it considers simultaneously hundreds of thousands of co-existing SNPs inside hundreds of genes and takes into account such parameters as meiotic recombination rate, selection pressure, population structure, etc.

All in all, this large-scale bioinformatics examination suggests that modern populations were formed by the admixture of two ancestral lineages that separated from each other around one million years ago and re-admixed around 0.3–0.1 million years ago.

## Methods

Genotype datasets for all the human chromosomes of the 1092 human genomes were downloaded from the 1000 genomes ftp site (ftp://ftp-trace.ncbi.nih.gov/1000genomes/ftp/release/20110521/) as Variant Call Format (VCF) files version 4.1 [22]. This database contains a total of 38.2 M SNPs, 3.9 M short indels and 14 K deletions for all the human chromosomes that have been used in this study. Information about parental haplotypes has been taken directly from Phase 1 of 1000 Genomes Project, since its genomic sequences were entirely “phased”. Ancestral/Derived status for every GVs was obtained from the “AA=” field inside column 8 of the 1000 Genome VCF files.

For the archaic Neanderthal genome sequence we used Denisovan genomic datasets from the Max Plank Institute for Evolutionary Biology that are available through public ftp site http://cdna.eva.mpg.de/denisova/VCF/hg19_1000g/(23). These Denisovan Variant Call Format (VCF) files contained coverage of the genome that is fairly uniform with 99.93% of the ‘mappable’ positions covered by at least one, 99.43% by at least ten, and 92.93% by at least 20 independent DNA sequences [23]. We computationally processed the Denisovan VCF files with our novel Perl scripts (Denisova_Haplo_Find.pl, Deni_Stat_generator.pl), which are available from the Additional file 1: SD1 on our web page (http://bpg.utoledo.edu/~afedorov/lab/YinYang.html).

All haplotypes of 1092 individuals were obtained with our pipeline of eight Perl programs (*HaploFind.pl; HapGroupGenerator.pl, MosaicStatGenerator1.pl, MosaicStatGenerator2.pl, MosaicStatGenerator3.pl, YinYangStatExplorer.pl, MosaicStatExplorer.pl, CombineStatsYY_Mos.pl, AncestralHapMatchFinder.pl*). A thirty-five page instruction manual for these programs with comments and notes is provided as a supplementary file. The program *HaploFind.pl* extracts the GVs having minor allele frequency >0.25 and constructs haplotypes with 50 adjacent frequent GVs. *HapGroupGenerator.pl* compares 2184 haplotypes from the 1092 individuals and builds haplotype groups that contain haplotypes having < =2 differences between them. Groups with > =100 occurrences are classified as common haplotypes (CHs). Other six programs have been used to compute different statistics for Yin, Yang and Mosaic CHs. The program *Ancestral_Hap_Match_Finder.pl* extracts the ancestral haplotype for each segment and finds its matches from the 2184 haplotypes in the 1000 Genomes populations in the corresponding segment.

Detailed description and scripts of all our Perl programs, their instruction manuals, the command lines for execution of programs, and examples of output files can be found in the Additional files 2, 6, 7, 8, 9 and 10.

In addition, all our programs are freely available from our website (http://bpg.utoledo.edu/~afedorov/lab/YinYang.html). The entire dataset of all haplotypes for each 5398 chromosomal segments generated by our programs is also available from this web site.

Computational simulations for the analysis of distribution and arrangement of SNPs in the population of virtual individuals were performed with our computational resource GEMA (Genome Evolution with Matrix Algorithms), which has been described by Qiu and co-authors [20]. In these simulations we varied the size of the population (*N*); the selection pressure (number of offspring per individual - *α*); and the number of recombination events during the gametogenesis in the genomes of virtual individuals (*r*). The program code and instruction manual for GEMA are available from web site (http://bpg.utoledo.edu/~afedorov/lab/GEMA.html) and from the original publication [20]. All SNPs generated during GEMA simulations were processed with the pipeline of Perl programs (*GemaBackupA_Process.pl, YinYangGema.pl, GemaSegments.pl, GemaHaplotypes.pl, GemaHapGroupGenerator.pl, Gema_HapGrouping.pl*). Perl scripts for these six programs, command lines for their execution, and their instruction manuals can be found in the Additional file 2 and in our website (http://bpg.utoledo.edu/~afedorov/lab/YinYang.html).

Statistics. *P*-values have been calculated using chi-squared test within Microsoft Excel package.

## Results

### Common Haplotypes (CHs)

**Fig. 1:**
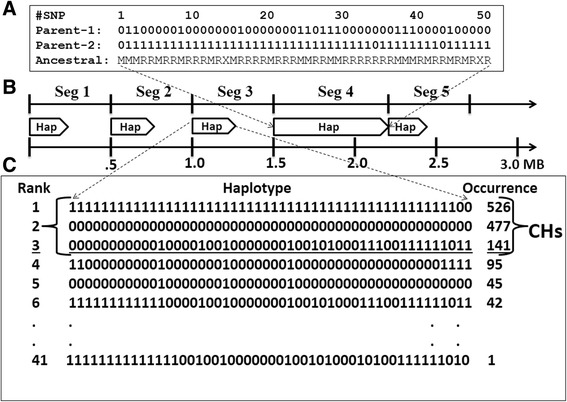
Haplotype construction and characterization. **a** Example of two parental haplotypes from segment 12 on chromosome 4 of CEU_NA07357 individual from 1000 Genomes. Following the 1000 Genomes Project, “0” means the presence of a reference allele, while “1” means an alternative allele in the haplotype. Only frequent GVs (with minor allele frequency >25%) have been used to construct haplotypes. In the last “Ancestral” line, “R” means that the reference allele is ancestral, “M” means that alternative (mutant) allele is ancestral, and “X” means unknown ancestral/derived status for the GV in the 1000 Genomes dataset. Information about every GV (identifier, location, alleles) and every haplotype are available from the Additional file 1: SD1. **b** Chromosomes have been divided into segments of equal length (500 Kb). From the beginning of each segment, 50 adjacent high-frequency GVs have been selected for construction of the haplotypes. When less than 50 frequent GVs were present inside the segment, this segment was elongated until a full-length haplotype with 50 GVs was complete (see Seg 4). **c** All haplotypes within a segment from 1092 individuals were grouped and ranked by the number of occurrences. Haplotypes that had been counted 100 or more times were named as common haplotypes (CHs). On 1b three common haplotypes exist and are shown above the solid line

All human autosomes have been divided into 500 Kb segments that are uniformly separated from each other as illustrated in Fig. 1. For each chromosomal segment, we studied haplotypes built from 50 adjacent GVs occurring with high frequency in modern humans (which Minor Allele Frequency (MAF) was >25% among 1092 sequenced genomes). Under this consideration, the physical length of haplotypes becomes a variable and depends on the density of frequent GVs in the locus under investigation. The invariable quantity of 50 frequent GVs in each haplotype allowed us to make a fair comparison of occurrences of haplotypes from different chromosomal locations. We chose 50 frequent GVs per haplotype because the average size of such haplotypes is around 60 Kb and it is congruent with the findings of Gabriel and co-authors who demonstrated that most of the human genome is contained in blocks/segments of substantial size and, within each segment, very few common haplotypes capture a vast majority (~90%) of the chromosomes in each population [24]. In our study, positions of chromosomal segments have not been aligned with positions of genes for the following reasons: i) positions of genes are distributed highly non-randomly along chromosomes; ii) the sizes of genes vary considerably from a few hundred up to two million nucleotides; iii) the beginnings of genes often have elevated GC-composition. Thus, our approach should present an unbiased view on the distribution of haplotypes of frequent GVs in the entire human genome.

Each of 1092 individuals from phase 1 of the 1000 Genomes Project is presented by two haplotypes that correspond to two parents of the individual. The presentation of haplotypes of examined individuals is exemplified in Fig. 1a. In addition to haplotype data, we extracted the ancestral/derived status for studied frequent GVs from the 1000 Genomes Project dataset (Fig. 1a). Occurrence of all haplotypes of 1092 individuals have been ranked and examined throughout the human genome as explained in Fig. 1c.

**Fig. 2:**
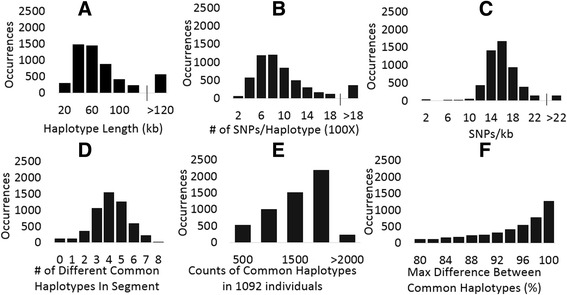
Properties of haplotypes of frequent GVs in the human genome. “Occurrences” on the vertical axis represents the number of segments that have specific characteristic shown on the horizontal axis. **a** Distribution of haplotype length (kb) in segments. **b** Distribution of total number of GVs per haplotype. Horizontal axis shown in multiplication by 100. **c** Density of all GVs in a haplotype. **d** Number of CH groups per segment. **e** Counts of all CHs in 1092 individuals in a segment. **f** Maximum allele differences between all CH groups within a segment

**Fig. 3:**
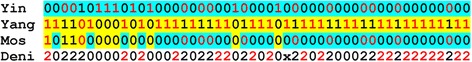
An example of Yin, Yang, and Mosaic haplotypes and a Denisovan diplotype from the segment 102 of chromosome 1. The alleles that match the human reference genome are shown as “0”, while the alternative alleles as “1”. The ancestral alleles are shown in black, and the derived ones are shown in red. Blue and yellow highlights demonstrate pieces of Yin (blue) and Yang (yellow) segments from which the Mosaic haplotype can be reconstructed. This Mosaic haplotype is constructed from 14 pieces and has 12 derived alleles. Ten Mosaic derived alleles (83%) match the Yin haplotype and only two Mosaic derived alleles match the Yang haplotype. The Denisovan diplotype is shown in the last row. For the diplotype “0” means that both parental alleles match the human reference genome, “2” means that both alleles match alternative alleles, “1” means that this ancestral Denisovan person is heterozygous at this particular allele, and “x” means that this allele is unresolved. The heterozygous status for our frequent GVs (MAF > 25%) is very rare for the Denisovan sequenced individual, so his homozygous diplotypes can be converted to haplotypes by the substitution of “2”s for “1”s

For each chromosomal segment, identical haplotypes from different individuals have been combined and ranked according to their occurrence among 1092 sequenced individuals. Nearly identical haplotypes (with 1 or 2 allele differences among 50 GVs) have been placed into the same group, which was assigned to the haplotype (“zeros/ones string”) with the highest occurrence. These haplotype groups are demonstrated in Fig. 1c and are available for each chromosomal segment from the Additional file 1: SD1. When a haplotype group was found 100 or more times among 1092 studied individuals, it was considered as a *Common Haplotype (CH)*. Distribution of CHs has been examined among all human autosomes in 5398 segments, and these data are shown in the Additional file 3 Table ST1. A subset of Table ST1 is shown in Table 1 to illustrate our approach. The basic features of CH occurrence and distribution are illustrated in Fig. 2. These data on CHs (number, size, occurrence) are congruent to the results of Gabriel and co-authors [24]. Visual examination of CH strings revealed that many segments contain mutually exclusive CHs that differ from each other at practically every polymorphic position. This phenomenon is seen in Fig. 2 F and also in Table 1, where the maximal difference of allelic variants between CHs from the same chromosomal segment is present in column 8. The first comprehensive examination of mutually exclusive CHs of humans was performed by [16]. The authors called these mutually exclusive haplotypes *Yin* and *Yang*, and we will follow their nomenclature here. We made a threshold of 47 or more differences among 50 polymorphic sites (> = 94% differences) to name a pair of CHs as Yin and Yang. This threshold was chosen to allow a few sequencing errors and/or occasional “jumping” of a particular GV from one haplotype into another, which occasionally happens in accordance to the Biased Gene Conversion (BGC) theory [25]. The example of Yin/Yang CHs are group 1 and 2 in Fig. 1C and Yin/Yang strings in Fig. 3. All in all, 56% of all segments (or 59% of segments that have two or more CHs) have Yin-Yang pairs of CHs. Since the abundance of Yin and Yang haplotypes was a big surprise to us as well as to Zhang and co-authors [16], we examined this phenomenon in detail.

**Table 1:** Distribution of Common Haplotypes in the Human Genome

CHR	Segment	Starting Position	Haplotype Length (KB)	Total SNPs in Haplotype	# Common Haplotypes	Occurrence of Common Haplotypes	Max Diff Common haplotypes	# Yin - Yang
CHR_1	1	30923	771	1382	1	166	NA	0
CHR_1	2	808223	44	649	1	348	NA	0
CHR_1	3	1302106	19	298	3	1847	47	2
CHR_1	4	1806647	53	719	4	1499	48	2
CHR_1	5	2302471	49	780	4	702	47	2
CHR_1	6	2802348	36	743	6	1308	43	0
CHR_1	7	3302745	24	439	5	1419	46	0
CHR_1	8	3803755	202	1022	1	121	NA	0
CHR_1	9	4302585	51	874	5	1595	49	2
CHR_1	10	4802513	28	511	6	1478	47	2
CHR_1	11	5302118	6	145	4	1692	46	0
CHR_1	12	5802376	79	1344	4	897	30	0
CHR_1	13	6302510	60	785	3	1278	49	2
CHR_1	14	6802171	24	332	4	1731	48	2
CHR_1	15	7302754	22	385	4	1492	43	0
CHR_1	16	7803891	52	731	7	1286	48	2
CHR_1	17	8304607	47	784	4	1411	49	4
CHR_1	18	8808185	119	1394	5	1592	47	2
CHR_1	19	9302942	34	664	5	1252	40	0
CHR_1	20	9814964	173	2423	3	703	10	0

This table presents segment-wise distribution of Common Haplotypes along the whole human genome. Common Haplotypes are defined as those which occur at least 100 times or more in the 1092 individuals. Segment length is the distance between the coordinates of the first and 50^th^ SNPs with frequency > =0.25. Starting position of each segment has been provided and segment length has been shown in kb. Haplotype pairs which differ in 47 or more loci (out of 50) have been defined as Yin-Yang haplotypes

### Characterization of Yin, Yang, and Mosaic CHs

**Fig. 4:**
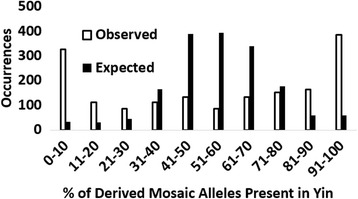
The observed data show 59% of cases where derived mosaic alleles primarily (> = 80%) came from either Yin or Yang. On the other hand, the expected dataset shows a normal distribution of Yin and Yang. The expected dataset was computationally created by randomly assigning each derived mosaic allele to Yin or Yang. In the observed data, 54.6% of mosaic derived alleles came from Yin, while 43.8% of mosaic derived alleles came from Yang. These percentages were used for the random assignment of derived mosaic alleles in the expected dataset

The highest occurrence of Yin and Yang CHs was detected by Zhang and coauthors (2003) when they considered haplotypes built from high-frequency GVs (MAF >20%). It dropped to about half when they reduced the MAF threshold to 5%. We also observed that MAF threshold influences the abundance of Yin/Yang pairs for our dataset (see Table 2). In the computations of Yin and Yang CHs by a pipeline of Perl programs, our algorithm assigns “Yin” to the CH with the highest occurrence, and “Yang” to its less frequent mutually exclusive counterpart. In addition to Yin and Yang, we also frequently observed CHs that could be reconstructed from many small pieces of Yin and Yang haplotypes (“zeros/ones” strings), as illustrated in Fig. 3. Every haplotype can be reconstructed from fragments of perfectly exclusive Yin and Yang strings, since they contain all possible allelic variants. When a reconstruction is achieved using only two or three large pieces, it can be explained by one or two recombination sites between Yin and Yang respectively. However, we frequently observed situations when reconstruction could only be achieved by combining 10–30 small pieces. We named these CHs, which can only be reconstructed from ≥12 pieces, *Mosaic* haplotypes. The origin of such Mosaic haplotypes is examined below. Our Perl program, *CountMosaics.pl* counts the minimal number of Yin and Yang “pieces” required to build a Mosaic haplotype. The characteristics of Yin, Yang, Mosaic, and all other CHs are present in the Additional file 4: Table ST2. Their parameters include numbers of ancestral and derived alleles in haplotypes and numbers of Yin-Yang “pieces” required for reconstruction of non-Yin/Yang CHs. An important consequence of this Additional file 4: Table ST2 is that the fraction of derived alleles in Mosaic haplotypes (average 31% of derived alleles) is considerably less than in Yin and Yang (averages of derived alleles 55% and 43% respectively). Moreover, the more “pieces” involved in the construction of Mosaic segments, the less derived alleles they have. For example, when we increased the threshold for Mosaic haplotypes to ≥20 pieces, the percentage of derived alleles in them was reduced to 24%. In addition, in most of the cases, the derived alleles of a Mosaic haplotype predominantly matched only one Yin or Yang haplotype from this mutually exclusive pair. For example, in the Fig. 3 ten out of twelve derived alleles of this Mosaic haplotype are found in Yin and only two derived alleles from this Mosaic haplotype are found in the Yang haplotype. Computations of all segments with Yin, Yang, and Mosaic CHs demonstrated that 59% of segments have a majority (80%) of Mosaic derived alleles belonging to one of the CH from Yin/Yang pair (see Fig. 4). There is no statistical preference between Yin or Yang for the derived alleles of Mosaic to be matched to. All these observations may have a simple explanation if we assume that a Mosaic haplotype is an ancestral stage for the evolution of one of the Yin or Yang haplotypes. The alternative hypothesis that Mosaic is a product of multiple recombination events between Yin and Yang is not in line with these observations, because in this case one would expect to see a unimodal distribution of derived Mosaic alleles among Yin and Yang haplotypes (which should be close to “Expected” distribution in the Fig. 4).

**Table 2:** Abundance of segments with Yin, Yang and Mosaic CHs

MAF threshold	Total #Seg	#Seg with ≥2 CHs	#Seg with Yin/Yang	%Segs with Yin/Yang	# Seg with Mosaic CHs
>0.1	5425	5259	491	9	228
>0.2	5408	5174	2125	39	1278
>0.25	5398	5097	3024	56	1720
>0.3	5380	4946	3622	67	1574

### Continental distribution of Yin, Yang, and Mosaic haplotypes

**Fig. 5:**
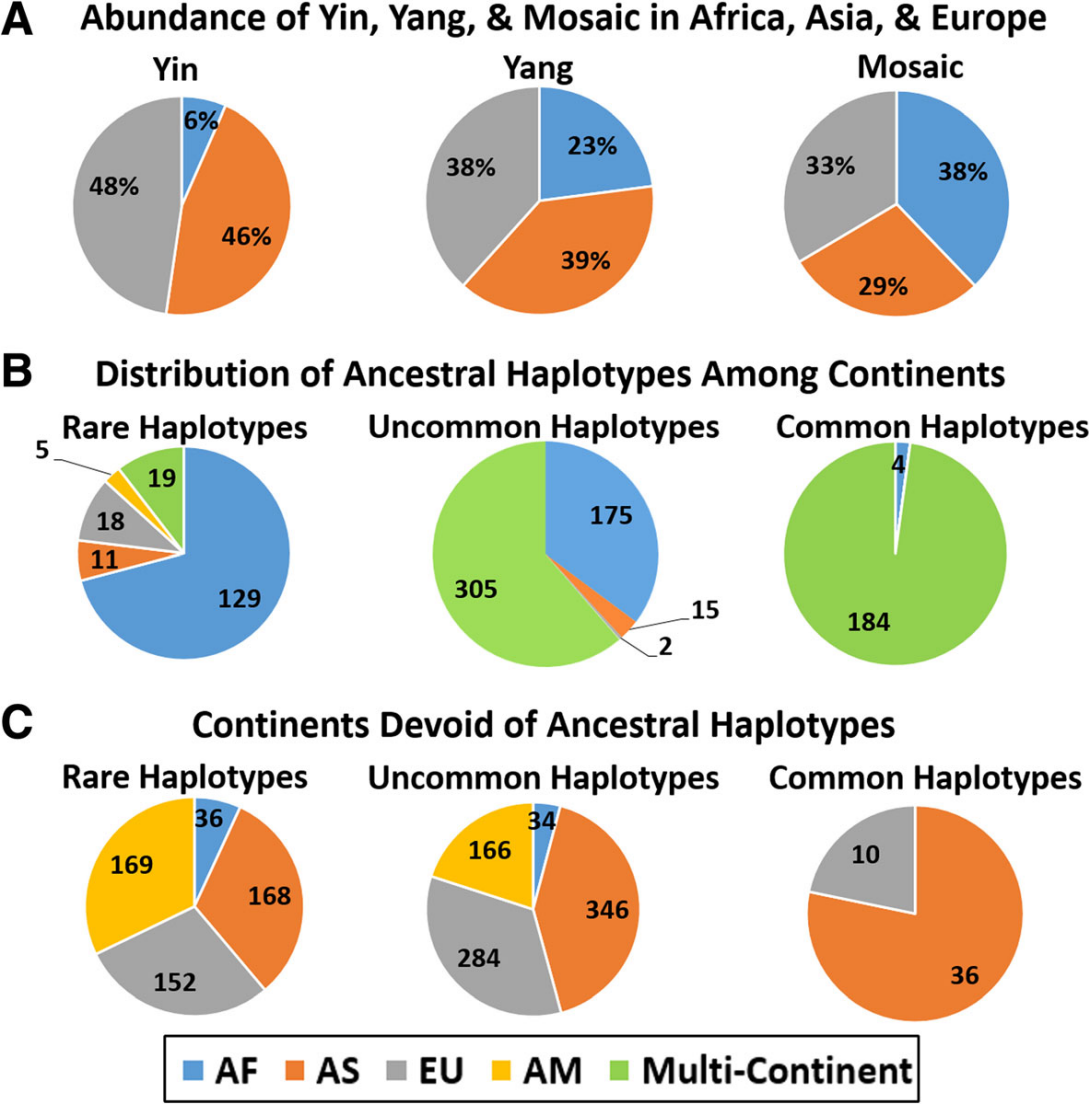
**a** Predominant occurrence of the Common Haplotypes (Yin, Yang, and Mosaic) among the African, Asian, and European populations. Occurrences of Yin, Yang, and Mosaic haplotypes were computed on each continent and then normalized (see M & M) to account for the uneven population sizes from the different continents. Predominance was determined by the highest normalized occurrence of the respective common haplotype in a segment. **b** and **c** Abundance of ancestral haplotypes in the continents. Rare, uncommon, and common haplotypes were determined by the number of matches to the ancestral haplotype out of 1092 individuals in a segment. Rare was classified as an ancestral haplotype, with only 1–3 matches in the segment, Uncommon was classified as 4–100 matches, and common was >100 matches. **b** For continent specificity, uncommon and rare haplotypes were defined as continent-specific if >90% of matches were found in a specific continent. Rare haplotypes were defined as continent-specific if 100% of matches were found in a specific continent. Multi-Continent means there was no continent specificity and matches to the ancestral haplotype were found on two or more continents. **c** Figure B represents the continents where ancestral haplotypes are absent (shows less than 1% match) for all the three types of haplotypes i.e. Rare, Uncommon, and Common haplotypes

Distribution of Yin, Yang, and Mosaic haplotypes among continents has been examined and the results are shown in Fig. 5. In our computations we name the most abundant haplotype as Yin and the least abundant as Yang. Fig. 5 displays that Yin haplotypes have statistically significant avoidance (*p* < 4*10^−6^, chi-squared test) of the African continent. Yang haplotypes as well have the same trend of minimal occurrence in Africa, though this is not statistically significant (*p* = 0.27). Both Yin and Yang are nearly equally abundant in Europe and Asia. At the same time, Mosaic haplotypes are slightly more abundant in Africa than in Europe and Asia. This non-random occurrence among continents strengthens the possibility that Yin and Yang may correspond to two ancestral lineages, as one out of two alternative hypotheses Zhang and co-authors initially suggested [16]. In an attempt to reconstruct these human ancestral lineages, we used Machine Learning approaches such as K-means Clustering and Decision Tree Classifiers to characterize the clusters that may correspond to these hypothetical lineages. Weka [26] and Rapid Miner [27] web computational resources were used for this purpose. Five normalized parameters for Yin and Yang haplotypes for each segment (total haplotype occurrence; the number of derived alleles; percentage of haplotype occurrence in Africa, Asia, and Europe) have been studied. However, despite our repeated attempts, we were unable to obtain any significantly well-separated clusters for these mutually exclusive haplotypes. These results are not shown here; details are provided in the Additional file 5: SD2.

### Comparison of Yin, Yang, and Mosaic with ancestral haplotypes

To evaluate the separation time of Yin, Yang, and Mosaic haplotypes we compared them with the available archaic human genome of one of the Neanderthal lineages (“pinky” Denisovan, [23]) whose DNA has been perfectly characterized (>30x coverage combined with high-quality reads in “bam” file). Alleles of frequent GVs that comprise our studied Yin, Yang, and Mosaic haplotypes of modern humans have been evaluated in the Denisovan genome sequence. Using these alleles, the Denisovan diplotypes have been assembled (parental haplotypes are not phased for this ancestral genome, so the diplotype is the only option). An example of this diplotype is shown in Fig. 3. In total, we computationally processed 1720 chromosomal segments that contained Yin, Yang, and Mosaic haplotypes and added to their files corresponding Denisovan diplotypes. This information on Denisovan diplotypes is available from our Additional file 1: SD1. It appears that the analyzed frequent GVs in the Denisovan genome were predominantly homozygous (>99%). This phenomenon simplified the comparison of the ancestral diplotypes with modern haplotypes, because in a vast majority of cases a diplotype is the summation of two identical copies of homozygous haplotypes (see Fig. 3). The computation of 1720 segments demonstrated that, on average, Denisovan haplotypes have the least number of derived alleles (18.0%), while Mosaic counterparts have 31% derived alleles, Yin – 55% and Yang 43%. Denisovan haplotypes are nearly identical (<=2 differences) to Mosaic haplotypes in 14% of analyzed segments (240 cases), whereas, such similarities with Yin and Yang haplotypes were found in 1.4% (25 cases) and 4.1% (71 cases) segments accordingly. Average allele difference between Denisovan haplotype and human CHs was also found to be least for the Mosaic haplotypes (12 differences on average), followed by Yang (19 differences on average) and Yin (25 differences on average). All these data indicate that the Denisovan haplotypes are most closely related to Mosaic haplotypes. Since the Denisovan haplotypes contain considerably fewer derived alleles than Mosaics (on average 18% versus 31% of derived alleles respectively), the Neanderthal people must have separated from modern humans earlier than the formation time of Mosaic haplotypes.

### Distribution of ancestral haplotypes among modern humans

Since the Denisovan haplotypes contain only 18% of derived alleles and 82% of ancestral ones, we were intrigued whether some modern humans still have completely “ancestral” haplotypes built exclusively from ancestral alleles of frequent GVs. To answer this question, a 100% ancestral haplotype of the same 50 GVs for each of 5398 segments have been deduced and compared with all available haplotypes of 1092 people. We allowed only one or two differences between the real haplotypes and the deduced 100% ancestral one to name them “ancestral”. Within 867 out of 5398 segments, ancestral haplotypes were found among modern humans. Within 182 segments we counted less than 4 ancestral haplotypes among all individuals (rare ancestral haplotypes on Fig. 5b); in 497 segments we counted from 4 to 99 ancestral haplotypes (uncommon ancestral haplotypes on Fig. 5b); and in 188 segments ancestral haplotypes were common (≥100 occurrences among 1092 people). The abundance of these ancestral haplotypes among continents have been computed and presented on Fig. 5.

Fig. 5 reveals that ancestral haplotypes are most abundant in Africa. For 188 segments where ancestral haplotypes are also the common ones (occurred ≥100 times) a majority of them, 184 segments, were observed on all continents and only four predominantly in Africa. However, these 184 “mixed” ancestral haplotypes still have the highest representation in Africa (42%), then in America (21%), Europe (20%), and Asia (17%).

### Modeling the appearance and abundance of CHs using GEMA computer simulations

Zhang and coauthors (2003) proposed that Yin-Yang haplotypes could arise due to the admixture of two ancient lineages of hominoids well before “Out-of-Africa” exodus or, alternatively, spontaneously from the sole ancestral population. The authors supported the latter hypothesis with computer simulations. However, Zhang et al. used simple simulations that did not take into account parameters that notably influence SNP dynamics and linkage. Therefore, to understand the origin of numerous mutually exclusive CHs we performed advanced computer simulations using our GEMA computational resource [20, 21]. The GEMA program generates a population of virtual individuals, creates an influx of novel mutations in their genomes and starts multiple cycles of individuals’ mating, offspring creations followed by their selection for surviving into the next generation. GEMA simulates dynamics of mutations under conditions close to natural. In these computations, we explored how the following parameters influence the formation of CHs and Yin/Yang pairs: 1) population size [*N* individuals per generation were changed in different simulations in the following range: 124, 250, 500, 1000, and 2000]; 2) number of meiotic recombination events per gamete (*r*) [*r* was either 48 events (average for humans) or 24, 12, and 6 recombinations]; 3) selection pressure [*α* parameter -- number of offspring per individual, which we changed from 2 (no selection) up to 10, which was the strongest in our experiments]. Other parameters were invariant and we used their default values: 1) flow of novel mutations per gamete [*μ* = 20, which was close to the natural rate of 20–50 novel mutations in human gametes]. 2) Mating schemes: random permanent pairs. 3) Co-dominant effect for ancestral/derived alleles (dominance coefficient: *h* = 0.5). 4) Distribution of mutation effects was *Experiment-C* (81% slightly deleterious; 9% beneficial; 10% neutral mutations). The results of our computer simulations are summarized in the Table 3.

**Table 3:** Dynamics and arrangement of SNPs in GEMA simulations

*Parameters*			*R e s u l t s*			
*N*	*r*	*α*	# SNP x 10^3^	#Freq SNP	% seg CHs	% seg Y/Y
124	48	5	50	6070	85	6.9
250	48	2	270	42333	98	12.5
250	48	3	146	16092	82	4.6
250	48	5	103	9644	73	5.5
250	48	10	80	6682	65	4.9
250	24	5	93	7045	96	25.4
250	12	5	82	5255	99	49.2
250	6	5	73	3863	100	69.0
500	48	5	193	12754	49	3.0
500	48	10	160	9109	55	2.6
1000	48	5	407	17724	35	2.0
2000	48	5	802	24897	25	1.1

This table presents an overall summary of the investigated chromosomal segments resulting from the analyses performed with different sets of SNPs according to their minor allele frequency (MAF threshold, shown in column 1). While column 2 shows total number of segments obtained in each experiment (see M&M for illustration), columns 3, 4, and 6 presents the number of segments with CHs, Yin/Yang haplotypes and Mosaic haplotypes respectively. Column 5 gives the percentage of total segments having Yin/Yang haplotypes

Column four represents the total number of SNPs in the population of virtual individuals. Column five demonstrates the number of frequent SNPs (MAF >25%) in the same modeling population. Column six represents percentages of segments that have one or more CHs (with frequency > =5% in the modeling population), while last column – percentages of segments with Yin/Yang CHs

**Fig. 6:**
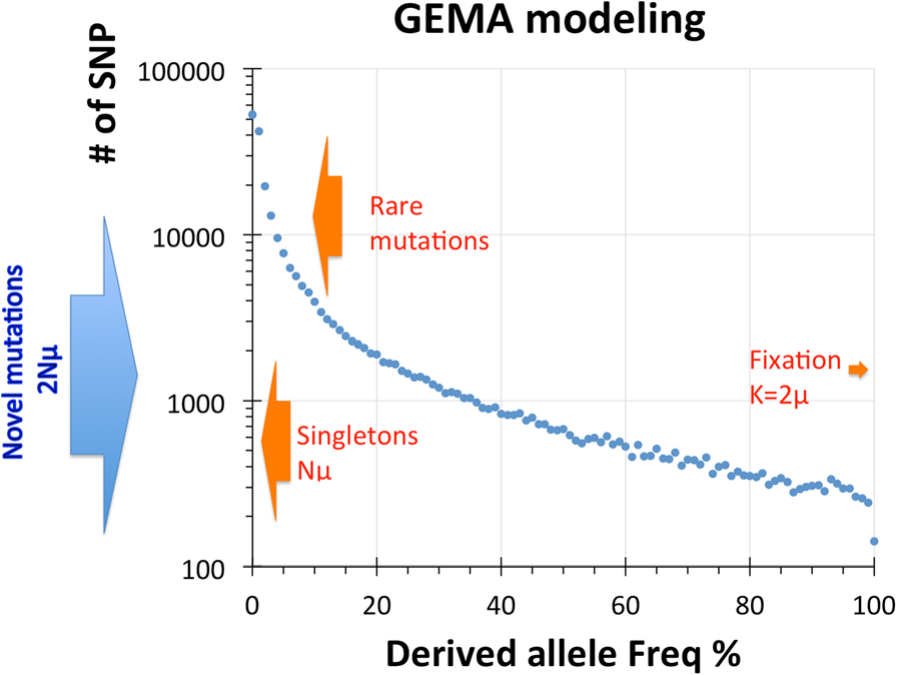
Distribution of derived SNPs in GEMA simulations. Parameters for this computation were the following: M = 20; a = 2; N = 250; r = 48; h = 0.5. Blue dots represent number of derived SNPs in the range of 1%

**Fig. 7:**
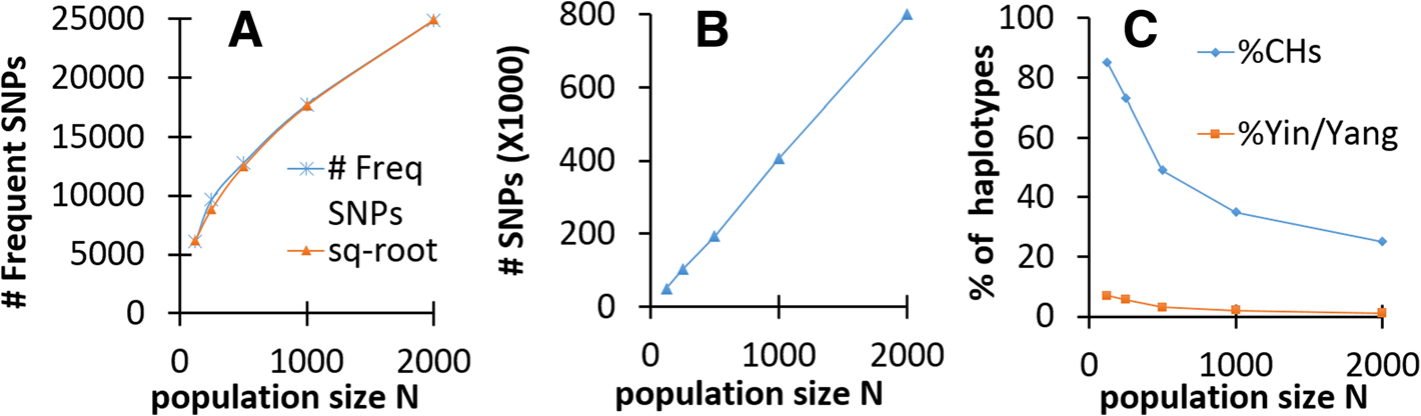
Dependence of SNP number and CH occurrence on the population size (N) in GEMA experiments. **a** Frequent SNPs with MAF > 25% are shown as blue stars. Red triangles show a square-root curve *c(N)* ^0.5^, where *c* is a constant that approximates the GEMA modeling data (c = 557). **b** Number of all SNPs (rare and frequent) in the modeling populations. **c** Percentage of segments that contain one or more CHs (blue line) and Yin/Yang pairs of CHs (red line)

In the GEMA simulations we first assessed the distribution of derived alleles by their frequency. A typical picture of such distribution is shown in Fig. 6. The highest abundance was always observed for very rare derived alleles and the lowest abundance for nearly-fixed derived alleles. The curve in Fig. 6 has the same shape as the real distribution of SNPs occurrence documented for the 1000 Genomes Project (see Extended Data Fig. 3 in [28]). In GEMA simulations and also in reality, the influx of novel mutations per generation is in direct proportion to the size of population *N* and equals *2Nμ* (blue arrow in Fig. 6). For the constant size population, approximately 50% of novel mutations transiently exist in a single copy per generation (singletons) and are removed in the next generation or in a few generations after their arrival (bottom red arrow in Fig. 6). Also, a considerable fraction of novel mutations exists in a few copies and still will drift away after several generations. Only a very minor fraction of derived mutations will survive and be fixed. The rate of fixed mutations per generation is *k = 2 μ* according to the Kimura’s law, which does not depend on the size of the population *N* [29]. [In several textbooks *μ* is the number of novel mutations per person and so *k = μ*.] Therefore, the number of frequent SNPs (MAF >25%) will be between these two extremes (*2Nμ* and *2 μ*) and will grow with the increasing size of the population, approximately as square root of population size, *N* ^*1/2*^ according to GEMA simulations (see Fig. 7a). In 1092 sequenced human genomes the number of frequent GVs (MAF >25%) is considerable and equals 2,944,337. Our simulation experiments with the parameters approximated to nature (*α* =5; *r* = 48, *μ* = 20) demonstrated that such high number of frequent SNPs in a sole population is achieved when *N* is about 25 million (see Table 3 and Fig. 7). In these computations we used the lowest estimations of novel mutations for human gametes *μ* = 20. If we use the highest evaluation *μ* = 50, then the size of the population for which number of frequent SNPs is 2.9 million dropped to 10 million people. Since all GEMA simulations gave equal chances for all virtual individuals in mating schemes and the number of offspring was the same for each virtual individual, the size of the population should be equal to the effective size *N* = *N*_*eff*_. In several independent estimations of *N*_*eff*_ for humans, this number is around 10^4^, which is strikingly lower than 10^7^ [30–32]. These estimations of effective population size are supported by a well-known formula for genetic diversity (*θ*), which shows that *θ = 4μN*_*eff*_ [33, 34]. The genetic diversity between European and/or Asian individuals is around 4x10^6^ [9]. Using *μ* = 100 for the number of novel SNPs per individual in the formula above, the value of *N*_*eff*_ becomes 10^4^. Since everybody agrees that population size of modern humans is much higher than archaic humans, it is unlikely that numerous frequent SNPs arrived from the sole ancestral population, which effective size must be around 10 million. An alternative scenario for the creation of multiple frequent SNPs is the admixture of subpopulations that were separated for hundreds of thousands of years (see Discussion).

We examined the abundance of CHs and mutually exclusive Yin-Yang pairs in the GEMA modeling under different conditions (Table 3). This table demonstrates that selection pressure (*α*), population size (*N*), and meiotic recombination rate (*r*) considerably influence the distribution of SNPs and formation of CHs in populations (see also Fig. 7). Many GEMA experiments demonstrate that Yin-Yang CHs are 5–10 times less abundant than in nature (compare Table 2 vs. Table 3). One of the most important parameters that stimulate the creation of abundant CHs and Yin/Yang pairs is the meiotic recombination rate (*r*), which should be low. On the other hand, the increase of the population size (*N*) causes a significant decrease of the abundance of CHs and the Yin/Yang pairs (Fig. 7c and Table 3). Due to the limitation of RAM in our Linux workstations, we were unable to increase the size of populations above *N* = 2000 in our computational modeling experiments. However if we extrapolate the results of our trends in Fig. 7a and Table 3, then for the *N* ≥ 1,000,000 there should be practically no CHs or Yin/Yang pairs. Therefore, our computer simulations demonstrate that the observation in modern humans of a high number of frequent SNPs (2.9x10^6^), together with an abundance of CHs (85% segments) and Yin/Yang pairs (56% of segments), could not originate from a single homogeneous population.

## Discussion

Humans possess 2,944,337 frequent GVs (MAF > 25%). This number is strikingly large. In order to get so many frequent GVs inside an isolated single population, its effective size should be around ten million, as demonstrated by GEMA modeling (see Fig. 7 and explanations in the Results). We also demonstrated that in modeling populations with large sizes, the arrival of mutually exclusive Yin/Yang CHs are very rare events. Since 56% of the investigated 5398 human loci have Yin/Yang CHs, special incidents must have happened during recent evolution to create these numerous mutually exclusive CHs. A straightforward possibility for the appearance of numerous Yin/Yang patterns is an admixture of two long-separated populations, which would also explain the observed large number of frequent GVs.

Let’s consider this hypothetical admixture and its consequences. According to Kimura’s law, a population has *k = 2 μ* fixed mutations per generation, which does not depend on the population size [29]. In humans, the value of *k* is around 100. In order to fix a million mutations, 10,000 generations are required, which roughly equals to 250,000 years (we assume 25 years per generation). Thus, after the admixture of two populations of comparable sizes that were separated from each other by 250,000 years, all mutations that had been fixed during their separation should become frequent GVs. So, this proposed admixture should automatically convert two million recently fixed mutations in both populations into frequent GVs, which, in addition, should be arranged as Yin/Yang CHs descended from two ancestral populations. (The actual number of frequent SNPs may be a little bit less if we assume that a fraction of the mutations that has been fixed are same in both populations.) These estimations demonstrate that the observed number and arrangement of 2.9x10^6^ frequent GVs in humans may have been created by a single “Great Admixture” of two major lineages that had been separated from each other around 400 thousand years. However, Yin/Yang pairs were observed only in 56% of the segments. In the rest segments one of the Yin or Yang might be lost due to selection (if one of them is more beneficial than the other). This process of CH loss reduces the number of frequent GVs, so the separation time of two ancestral populations might be up to 800 thousand years to allow creation of about 3 million frequent GVs after their admixture.

Modern humans are widely spread across the globe and adapted to a number of diverse environments on different continents. In general, an admixture of different groups of people from different places should be beneficial overall and allow new combinations of various adaptations. For example, a Neanderthal EPAS1 allele is widespread in Tibetans and helps living in high altitudes [35]. Other beneficial examples were recently reviewed by Haber and co-authors [36]. There were multiple well-known admixtures in recent human history, including peopling of New World by Europeans and Africans. Several admixtures of long-separated archaic human lineages are also described in the literature [36–38]. They include an admixture occurred between Neanderthal people and archaic humans [36, 38]. Importantly, this latter event did not create Yin/Yang CHs, since the number of Neanderthals at the admixture was negligible compared to archaic humans and, thus, Neanderthals’ recently fixed mutations were predominantly converted into rare GVs in modern humans. Recently David Curtis described and example of human Yin/Yang rare haplotype pair built from rare missense SNPs [18]. One of his plausible explanations of the origin of this locus was an admixture. For the conjectured “Great Admixture” of two ancestral populations named *A* and *B*, their sizes should differ from each other by no more than three times in order to generate Yin/Yang CHs. Because Yin CHs have strong avoidance of Africa, (see Fig. 5) it is reasonable to surmise that one of the *A* or *B* ancestral lineages should have evolved outside this continent and was a distant relative to the Neanderthals. At the same time, the prevalence of ancestral and Mosaic haplotypes in Africa supports the possibility that another ancestral lineage had likely developed inside this continent. In our hypothetical scenario, *A* and *B* ancestral lineages are the primary sources for Yin/Yang CHs. The observed Mosaic CHs may be interpreted as favorable combinations of mutations in one of the ancestral *A* or *B* populations that have been beneficial to people and, hence, have been preserved for hundreds of thousands of years in the ancestral populations.

**Fig. 8:**
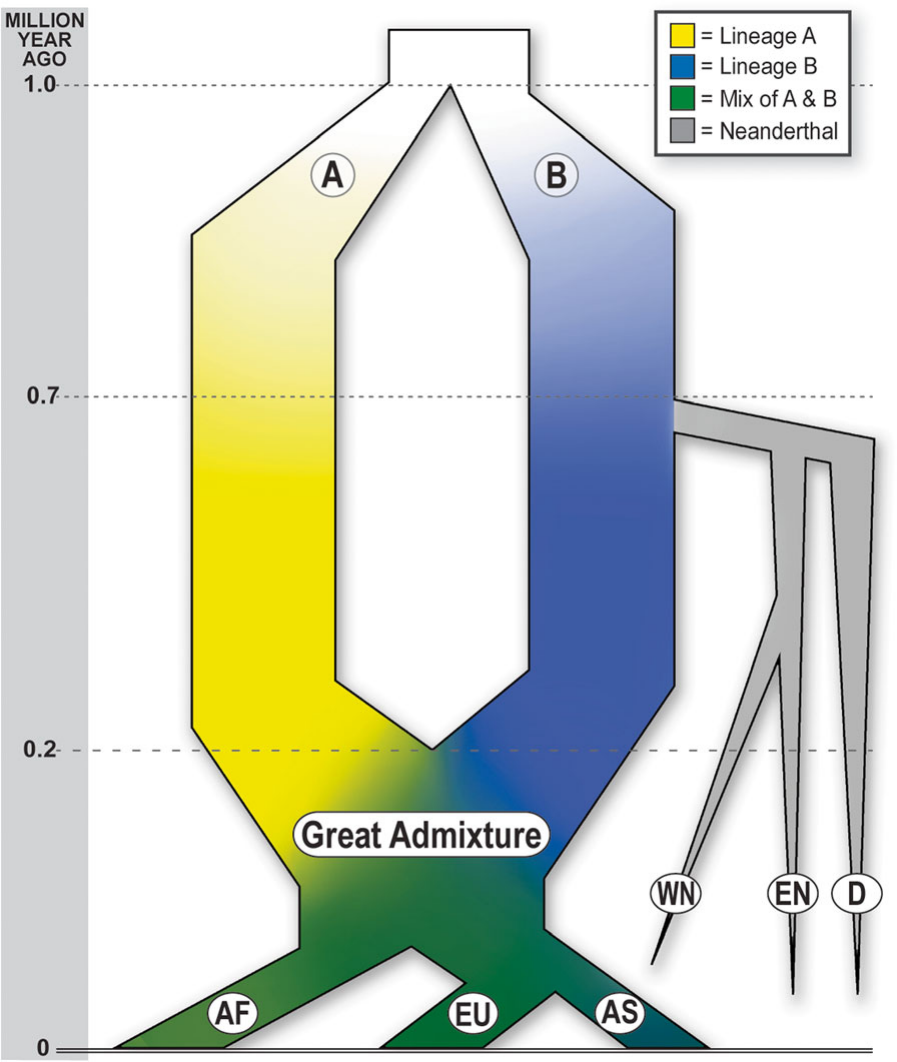
Scheme of the hypothetical Great Admixture event that led to formation of modern humans. Around 1 MYA two archaic lineages a and b separated from each other. These two lineages **a** and **b** are represented by the yellow and blue arms respectively. The gradient color scheme in these two arms shows appearance and gradual accumulation of novel GVs in each of the two lineages. The grey branches at 0.7 MYA time point shows the Neanderthal separation. WN, EN and D represents western Neanderthal, eastern Neanderthal and Denisovan respectively. The “Great Admixture” is represented by the appearance of the green color around the 0.2 MYA time point (right after the yellow and blue arms join each other). The modern populations are represented by the three descending branches following the “Great Admixture” event. AF, AS, and EU denotes African, Asian and European populations respectively

Is it possible to estimate the time of the hypothetical “Great Admixture” event? The Denisovan CHs give us a good reference point, which helps the assessment. The analyzed Denisovan CHs possess 18% of derived frequent alleles present in modern humans, while Yin/Yang pairs share on average 1% of derived alleles. Therefore, separation of two ancestral lineages *A* and *B* must have occurred prior to the separation of archaic humans with Neanderthals. At first approximation we assume that on average modern humans may have about 50% of derived alleles for frequent SNPs. The abundance of these derived alleles should be lower in the ancestral genomes. In our estimation, we assume a linear decline of the percentage of these derived alleles in time backwards. Taking the separation time of Neanderthals with modern humans to be about 0.7 MYA [between 0.8–0.55 Mya according to several independent assessments [23, 38] and also based on our finding of 18% of derived alleles for frequent GVs in the Denisovan genome, we estimated that the time of separation of A and B lineages should be 1.5 times older than the separation of Neanderthals and modern humans. Similarly, considering the fact that Mosaic haplotypes have on average 31% of derived alleles for frequent GVs, we estimated that the time period of Mosaic haplotypes’ formation was around 0.4 Mya. Our hypothetical scheme of the origin of modern humans from the Great Admixture event is illustrated in the Fig. 8. We conjecture that the “Great Admixture” occurred roughly 300–100 thousand years ago (0.2 MYA on average in the Fig. 8). In this illustration we draw the Neanderthal branches and the branches of modern African, Asian, and European populations based on Kuhlwilm and co-authors paper [39].

## Conclusions

Our results support the multi-regional theory of creation of modern people with multiple local admixtures with one “Great Admixture” event that generated a majority of frequent GVs and abundance of Yin/Yang CHs.

Dynamics and arrangement of GVs in modern humans represent very intricate patterns. Multiple parameters including selection pressure, meiotic recombination rates, and size of the population are very important in the analysis of these patterns. Advanced computer simulations, like GEMA, are extremely helpful in understanding SNP abundance and arrangement at the genomic scale.

## Additional files

Additional file 1: SD1. Complete set of our pipeline of Perl programs to characterize human haplotypes and Denisovan diplotypes is presented in this file. All the output files which contain information about every GV with MAF >0.25 (identifier, location, alleles) and every haplotype in the human genome and every diplotype in the Denisovan genome are also available in this file. The file is archived and compressed using UNIX *gzip* and *tar* commands. The size of this file is 2.8 GB and it is available from our web-page: (http://bpg.utoledo.edu/~afedorov/YinYang.html). (GZ 2.63 gb)

Additional file 2: Instruction Manual. Instruction manual and protocols for Perl programs for construction and analysis of haplotypes of frequent genetic variants and protocol for GEMA modelling. The file name is InstructionManualYinYang.docx in our website. (DOCX 685 kb)

Additional file 3: Table ST1. Distribution of CHs has been examined among all human autosomes in 5398 segments, and these data are shown in the Additional file 2: Table ST1. (XLSX 643 kb)

Additional file 4: Complete table presenting the characteristics of Yin, Yang, Mosaic, and all other CHs in all the 22 human autosomes. (XLSX 1667 kb)

Additional file 5: SD2. The results of Machine Learning approaches (Weka, (26) and Rapid Miner, (27) web computational resources) are presented here. (DOCX 238 kb)

Additional file 6: Table S2. A prototype of one of the output files generated in Step II. The name of the file is CORE_HAPS_New_140 which is for chromosome 1 segment 140. The file is transferred from UNIX to pc environment in MS Excel format. (XLSX 23 kb)

Additional file 7: Table S3. A prototype of one of the output files generated in Step II. The name of the file is STAT_FOR_Yin_Yang_New_1 which is for chromosome 1. The file is transferred from UNIX to pc environment in MS Excel format. (XLSX 50 kb)

Additional file 8: Table S4. A prototype of one of the output files generated in Step II. The name of the file is STAT_FOR_Mos_New_1 which is for chromosome 1. The file is transferred from UNIX to pc environment in MS Excel format. (XLSX 98 kb)

Additional file 9: Table S5. A prototype of one of the output files generated in Step II. The name of the file is Combined_STATS_YY_Mos_1 which is the output file for chromosome 1. The file is transferred from UNIX to pc environment in MS Excel format. (XLSX 140 kb)

Additional file 10: Table S6. A prototype of one of the output files generated in Step V. The name of the file is CORE_HAPS_with_DENI_Pinky_10 which is the output file for chromosome 1 segment 10. The file is transferred from UNIX to pc environment in MS Excel format. (XLSX 21 kb)

## Abbreviations

BGC: Biased Gene Conversion
CH: Common haplotype
GEMA: Genome Evolution with Matrix Algorithms
GV: Genetic variants
IBD: Identical-by-descent
MAF: Minor allele frequencies
mt-DNA: Mitochondrial DNA
NGS: Next generation sequencing
NRY: Non-recombining region of Y chromosome
SNP: Single nucleotide polymorphism
VCF: Variant Call Format
vrGVs: Very rare genetic variants
