# Supplementary material for: Intricacies in arrangement of SNP haplotypes suggest “Great Admixture” that created modern humans
Source: BMC Genomics. 2017 Jun 5;18:433. doi: 10.1186/s12864-017-3776-5 (PMC5741169; doi:10.1186/s12864-017-3776-5)
Supplement: Supplementary file 2 — Instruction manual and protocols for Perl programs for construction and analysis of haplotypes of frequent genetic variants and protocol for GEMA modelling. The file name is InstructionManualYinYang.docx in our website. (DOCX 685 kb) [file 12864_2017_3776_MOESM2_ESM.docx]

**Instruction manual for Perl programs for construction and analysis of haplotypes of frequent genetic variants.**

**Human Chromosomes and 1092 Individuals:**

The 1000 Genome Phase I database contains SNP data for all 22 human autosomes and the ‘X’ chromosome for each of the 1092 individuals from 14 different populations belonging to four different continents. Version 4.1 of this dataset contains a variety of genetic variants (GVs) - a total of 38.2M SNPs, 3.9M short indels and 14K deletions for all the human chromosomes. Since the majority of the GVs are SNPs, here we will use the word SNP for all GVs.

**The 1000Genomes data files in INTRON2:**

The 1000Genomes data files are stored in a specific directory of the supercomputer intron2 workstation (refer figure 1 below).


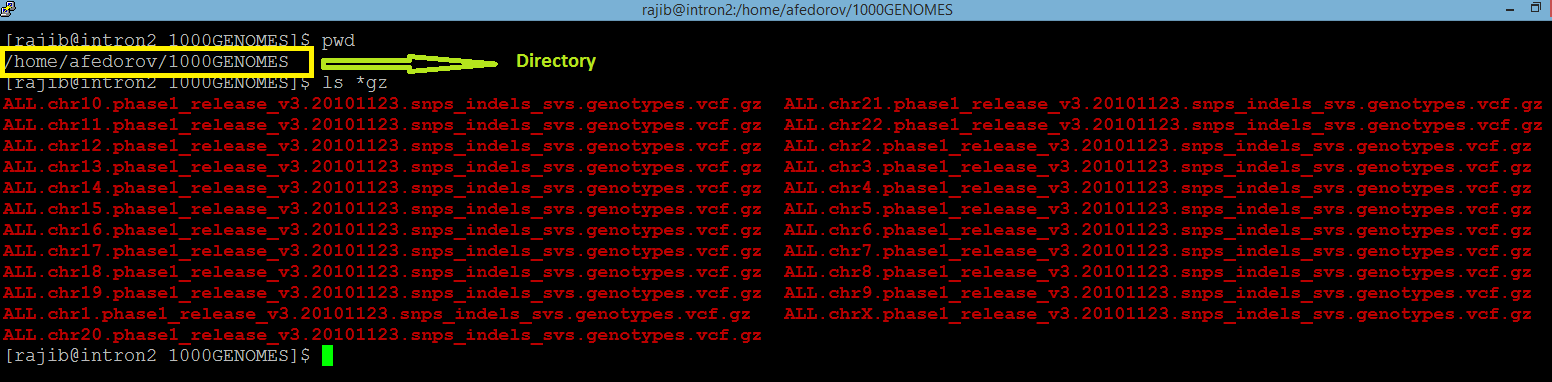


**Figure 1: screenshot of directory with 1000Genomes data files in the supercomputer intron2 workstation**

**STEP I: Extraction of SNPs and building the haplotypes:**

The perl script ***Haplo_find.pl*** opens and processes the 1000 Genomes .vcf.gz file for the desired chromosome. This program filters the SNPs with desired frequency and processes them into haplotypes. The user chooses the parameters (such as the chromosome of choice, desired frequency of SNPs, number of SNPs in the haplotype and a threshold count for defining the Common haplotypes) according to preferences in the arguments in the command line. For example, let us consider the following screenshot of a command line for executing ***Haplo_find.pl****:*


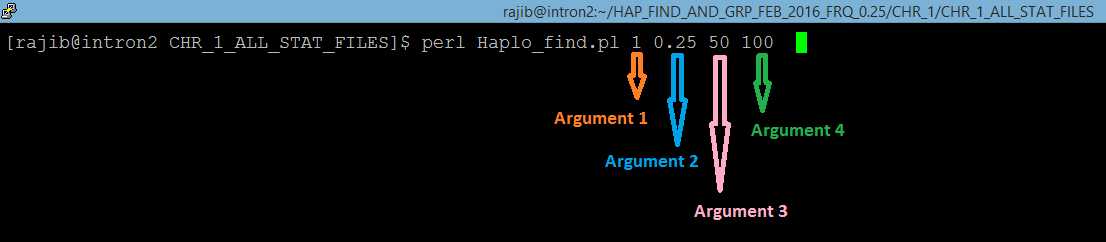


**Figure 2: screenshot of command line for execution of Haplo_Find.pl**

The command line in the figure 2 above computes the haplotypes for Chromosome **1 (argument 1)**, with SNPs having frequency >**0.25** **(argument 2)** for minor alleles (MAF). The program constructs haplotypes with **50 (argument 3)** such adjacent frequent SNPs. Common haplotypes will be those which occur >=**100 (argument 4)** times in the populations (1092 individuals).

While processing the .vcf.gz file for the intended chromosome, the script ***Haplo_find.pl*** considers the first SNP coordinate in the chromosome as the beginning of the first **SEGMENT**. In the 1000Genomes dataset, the SNP coordinate is in the second column under the header ‘POS’ and the SNP ID is in the 3^rd^ column with ‘ID’ header (see screenshots below). The program starts scanning the file for SNPs with user specified frequency (>0.25 MAF in the given example). The frequency is denoted as AF and provided in the INFO column (column #8, see figure 3 below).


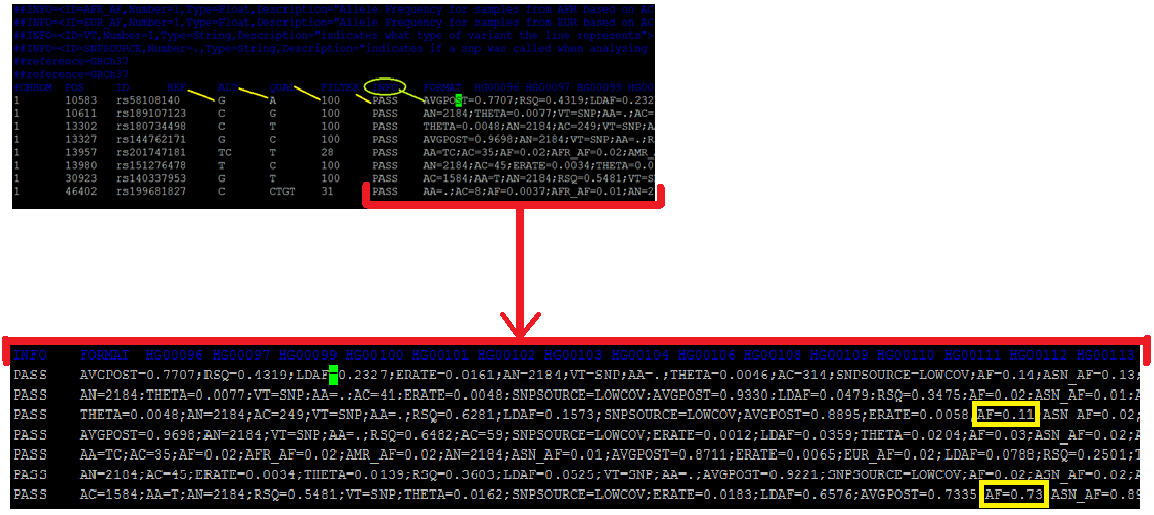


**Figure 3: screenshots of 1000Genomes vcf.gz file format**

When the program finds the first SNP with the desired frequency, it notes the coordinate and keeps looking for the desired SNPs until it finds the 50^th^ such SNP. When the 50^th^ SNP is found, the coordinate of the 50^th^ SNP is marked. The distance between the 1^st^ and the 50^th^ SNP is defined as the **HAPLOTYPE LENGTH**. If the HAPLOTYPE LENGTH is <= 500,000, the next segment starts from the coordinate of the 1^st^ SNP + 500000. If the HAPLOTYPE LENGTH is > 500,000, the starting coordinate of the next segment becomes (the 50^th^ SNP coordinate + 1).

The 50 lines corresponding to the 50 filtered SNPs, containing the general information and genotype information of the 1092 individuals are saved in output files named **ALL_STATS_Cx-y** (where x denotes the Chromosome number and y denotes the segment number). The genotype information of the 1092 individuals are then extracted by the program.

Genotype annotations for an individual homozygous with mutant genetic variants is annotated 1|1 in the VCF file. Homozygous presence of reference alleles in an individual is annotated 0|0. Genotype of heterozygous individual is annotated 0|1 or 1|0. These genotype information is available from the column respective to each individual identified by a unique identifier (HGxxxxxx or NAxxxxxx) in the header line. The figure 4 below shows these genotype information and Individual Identifiers from one of the .vcf.gz files. Since the 1000Genomes data is phased, we have denoted the first genotype as M (maternal) and the second as P (paternal) for ease of understanding, although practically it is not possible to identify the maternal and paternal chromosome with certainty from this data.


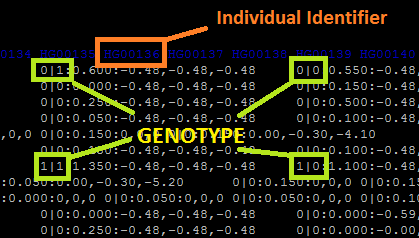


**Figure 4: screenshot of genotype annotation in one of the 1000Genomes data files**

The program Haplo_Find.pl extracts and stores these genotype information for each of the 1092 individuals for the 50 SNPs of interest (MAF >0.25) and constructs the two haplotypes (maternal and paternal) for each of the 1092 individuals. Practically, each haplotype is a string consisting of 50 single digits- 0s and 1s.


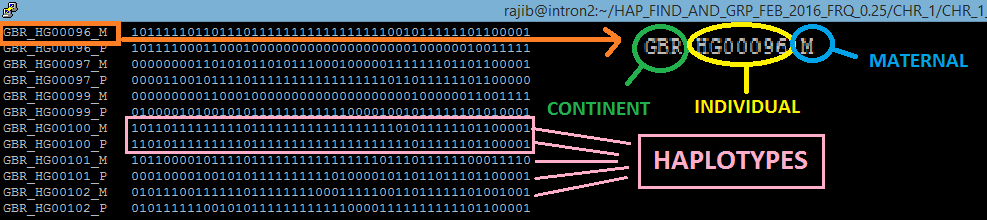


**Figure 5: screenshot showing haplotypes in one of the output files named ALL_STATS_Cx-y**

From the .vcf file, the program also extracts the individual identifier. The population each individual comes from is available from the 1000Genomes website. These two information are then combined together by the program to create a unique identifier along with M (for the maternal chromosome) and P (for the paternal chromosome). Thus, for example, in the figure 5 above, two chromosomal strands for the individual highlighted are denoted as GBR_HG00096_M and GBR_HG00096_P where GBR is the population and HG00096 is the individual identifier. M and P stands for maternal and paternal respectively.

The haplotypes are stored along with the respective identifiers in the output files named **ALL_STATS_Cx-y** (where x denotes the Chromosome number and y denotes the segment number).

Finally, the algorithm compares the 2184 haplotypes (strings of 0s and 1s as shown in the figure above, 2 * 1092 = 2184) with each other and counts the occurrences of the haplotypes. The haplotypes that occur more than the user specified threshold (in the example here >=100) are defined as Major haplotypes. These common haplotypes are listed at the bottom of the output file **ALL_STATS_Cx-y** (see figure 6 below).


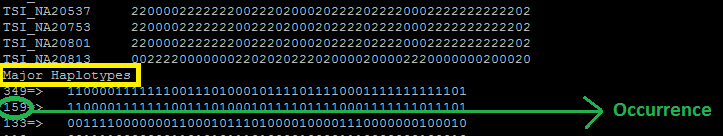


**Figure 6: screenshot showing Major haplotypes in one of the output files named ALL_STATS_Cx-y**

These processes continue for the entire length of the chromosome and the resulting segments, their haplotypes and other information are saved in automatically created output files for each respective segment.

This STEP requires about 1.5 hr to finish computation for Chromosome 2 which is one of the largest chromosomes and have the highest number of SNPs with frequency >0.25 (233,023). Therefore, for each of the other chromosomes, complete execution of this program requires less than 1.5 hr.

Upon execution, this program automatically generates the following output files:

Outputs:

1. **ALL_STATS_Cx-y** for all segments. For example:

**% vi ALL_STATS_C1-143**

This output file was created on Sun Mar 13 00:08:06 2016

CHR_1 Segment_143 Hap Length= 49259

Total no. of SNPs in this segment= 706

SNPs with freq > 0.25 & < 0.75 are:

1 71320836 rs34747258 GA G 442 PASS AA=GA;AC=605;AF=0.28;AFR_AF=0.22;AMR_AF=0.30;AN=2184;ASN_AF=0.20;AVGPOST=0.9943;ERATE=0.0006;EUR_AF=0.36;LDAF=0.2768;RSQ=0.98

1 71325044 rs1327460 T C 100 PASS ERATE=0.0004;AVGPOST=0.9985;AA=C;AC=1376;AN=2184;VT=SNP;LDAF=0.6295;THETA=0.0006;SNPSOURCE=LOWCOV;RSQ=0.9975;AF=0.63;ASN_AF=0

1. **STAT_Chr_1-SNP_50-HAP_100**

**% vi STAT_Chr_1-SNP_50-HAP_100**

This output was generated on Sat Mar 12 23:52:29 2016

#of_Major_HAPs Segment_Occurrence

0 70

1 47

2 53

3 90

4 96

5 67

6 20

7 11

8 2

1. **HAP_Length_C_x-SNP_50-HAP_100**

**% vi HAP_Length_C_1-SNP_50-HAP_100**

This output was generated on Sat Mar 12 23:52:29 2016

Seg_No Hap_L

1 771170

2 43840

3 19193

4 52649

5 49228

6 36368

7 23733

1. **No_of_Major_HAP_C_x-SNP_50-HAP_100**

**% vi No_of_Major_HAP_C_1-SNP_50-HAP_100**

This output was generated on Sat Mar 12 23:52:29 2016

Seg_No No_of_Maj_Hap

1 0

2 0

3 6

4 3

5 1

6 4

7 4

1. **TABLE_C_x-SNP_50-HAP_100**

**% vi TABLE_C_1-SNP_50-HAP_100**

Seg_No Hap_L No_Major_Hap Top_Hap_Ct 2nd_Hap_Ct 3rd_Hap_Ct

1 771170 0 11 11 10

2 43840 0 54 53 30

3 19193 6 548 227 198

4 52649 3 299 140 133

5 49228 1 133 97 55

6 36368 4 313 176 134

1. **TOP_HAP_CT_C_x-SNP_50-HAP_100**

Lists the integer value of **[Top Hap count in the segment/50].** The top hap in Seg 4 occurs 274 times. So for Seg 4, this file will have a value of (274/5) = 5.

**STEP II:YIN, YANG and MOSAIC Haplotypes**

1. **Grouping of nearly identical haplotypes into ‘Haplotype Groups’ and identifying the Common Haplotypes**

This step uses a system call (**system_for_Yin_Yang.pl)** to run the **Hap_Group_generator.pl** program for all the segments created for each chromosome in STEP I.

The command line for execution of this step is as follows:

**% perl system_for_Yin_Yang.pl**

The *Perl* script **Hap_Group_generator.pl** uses the files named **ALL_STATS_Cx-y** generated in step 1 as inputs for each segment. The program scans the input file for each segment and then compares the 2184 haplotypes (from the 1092 individuals) and identifies haplotypes which have <=2 differences between them. Such haplotypes are defined as a ‘**haplotype group’.** Such a haplotype group is represented by the haplotype that has the maximum occurrence within that group. The list of all members of a haplotype group is saved in the output file named **Hap_Grouping_New_x** (where x denotes the segment number) along with their respective occurrences in decreasing order (see figure 7 below). Note that the differences between the representative haplotype and other haplotypes in group 1 have been underscored in red in figure 7.


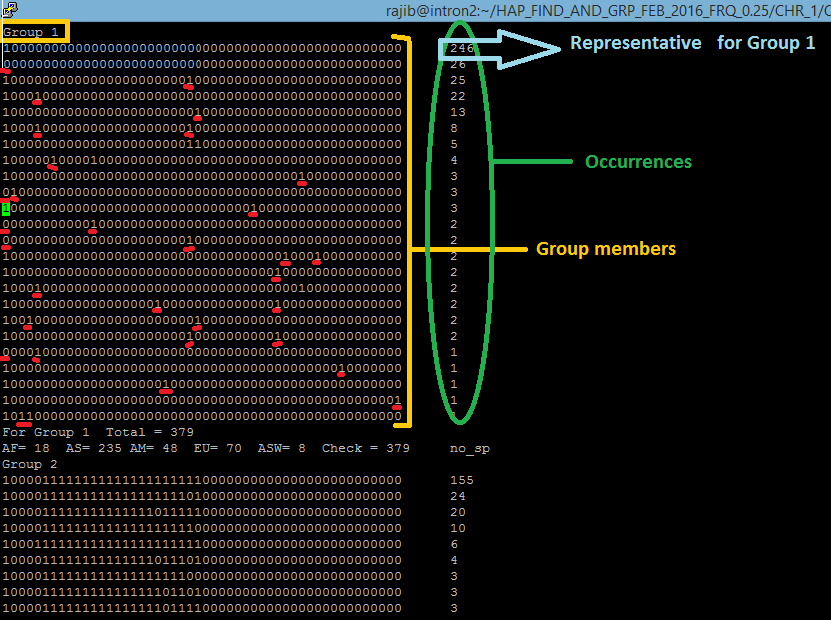


**Figure 7: screenshot showing grouping of haplotypes in one of the output files named Hap_Grouping_New_x**

The combined occurrence of all group members is considered as the group occurrence and is considered to define effective Common haplotypes. Thus, a haplotype which is not listed as a Major haplotype in the STEP I output file **ALL_STATS_Cx-y** (due to occurrence <100 times) could feature in the STEP II output file **Hap_Grouping_New_x** by virtue of additional occurrences of similar haplotypes (haplotype group members with 1 or 2 differences) leading to a combined total occurrence of >=100. For example, let us consider the following figure 8. In this case, the 1^st^ member of Group 5 occurs 71 times in the segment. Therefore, per our definition of Major haplotypes (occurrence >= 100 times), this haplotype is **NOT** a Major haplotype. So, the respective **ALL_STATS_Cx-y** file will not have this haplotype listed as a common haplotype. But as we find the other members of Group 5, their occurrences add up and increases the Group occurrence to 105. Therefore, Group 5 qualifies as a common haplotype. Since Group 5 will be represented by its 1^st^ member (with highest occurrence = 71), the first member now is considered a common haplotype.


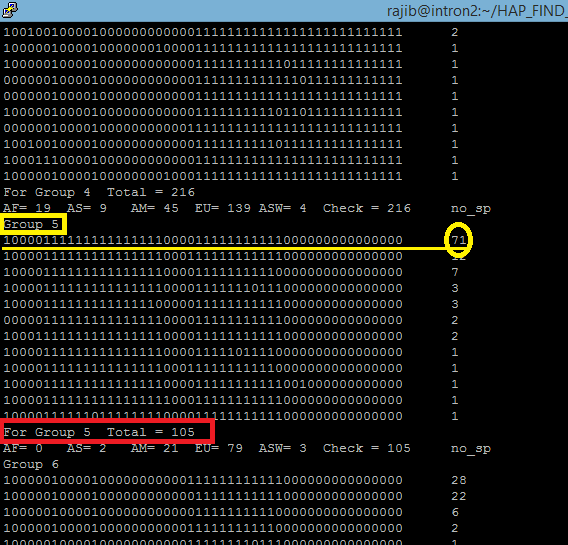


**Figure 8: screenshot showing grouping of haplotypes**

The program also translates the population information of the individuals into continent information and computes the continental distribution of the Common haplotype groups. In the figure above, AF denotes the African continent while AS and EU denotes Asia and Europe respectively. AMR denotes the America and ASW denotes ‘America South West’. If a haplotype group occurs predominantly (>=80%) times in one of these continents, it is defined as a ‘continent specific’ haplotype (for example, Asia specific haplotype and so on). Such continent specificity is noted as ‘AS_SP’ followed by the percentage of occurrence at the end of the continent information line (for example, **AS_SP 90%**). If predominant occurrence is not observed, the haplotype is considered to have ‘no continent specificity’ and this is noted as **‘no_sp’** at the end of the continent information line (see figure above). All the above information are saved in automatically generated output files named **Hap_Grouping_New_x,** where x denotes the segment number.

The common haplotypes are then listed and saved into a second set of automatically generated output files named **CORE_HAPS_New_x**, where x denotes the segment number. The continental distribution and the ancestral and derived allele percentage of the common haplotypes are also saved in the same output files.

R stands for ‘reference allele’ while M denotes mutant/alternative allele. The alleles for which ancestral information is not available information are represented by ‘x’ in the output file (for example, see the figures 7 and 8 above). Using the ancestral allele information available from the vcf.gz file, the algorithm deduces the ancestral state of each allele in each common haplotype. In the ancestral state, ‘A’ denotes ancestral allele and ‘D’ denotes derived alleles. The unknown ‘x’ positions are kept as ‘x’. Then, the program calculates the percentage of ancestral and derived alleles in each common haplotype. The loci denoted with ‘x’ are not considered while calculating the ancestral and derived allele percentage. These information are also stored into the **CORE_HAPS_New_x**, output files. Below is a screenshot of a portion of one such file.


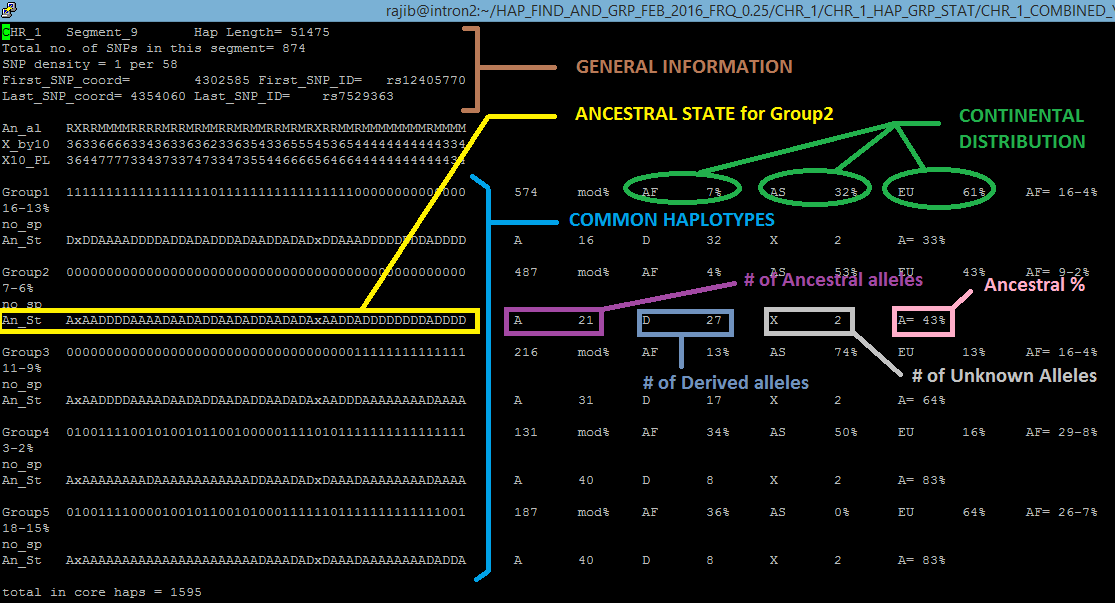


**Figure 9: screenshot of one prototype of output files named CORE_HAPS_New_x**

In addition to these information, the **CORE_HAPS_New_x** files also contain relevant general information about the segment (see the top of the file in figure 9).

1. **Identifying YIN, YANG and MOSAIC haplotypes**

Next, the algorithm compares the Common haplotype groups with each other and counts differences. If two haplotype groups are found to have >=47 differences between them, they are defined as a pair of YIN and YANG haplotypes. The common haplotype groups other than the YIN and YANG are considered as mosaics since these carry similarities to both YIN and YANG. For better clarity, a MOSAIC haplotype can be perceived as consisting multiple pieces from the YIN and YANG haplotypes. The number of differences between the common haplotype groups and the YIN, YANG, and MOSAIC haplotypes are listed in the files named **CORE_HAPS_New_x.** This is illustrated in the following figure 10. We can see that the # of differences between Group1 and Group3 is 49. Therefore, Group1 and Group3 are a YIN-YANG pair. Since the differences between all other common haplotype groups is less than 47, we have only one YIN-YANG pair in this segment. The program also computes the number of such pieces in a mosaic group by comparing it with the YIN and YANG at the same time.


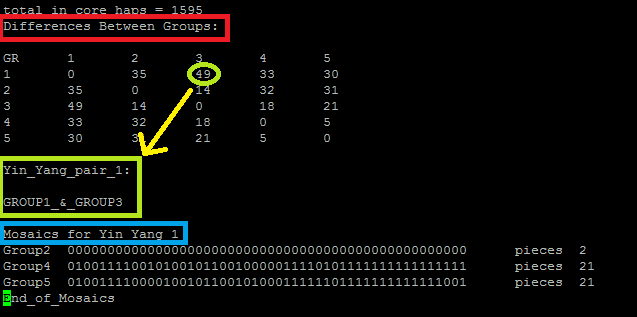


**Figure 10: screenshot showing a different portion of the output files named CORE_HAPS_New_x**

If a segment has more than one YIN-YANG pair, the program identifies all of them and also lists mosaics for each YIN-YANG pair separately. Number of pieces in a mosaic group is also computed in each case by comparing with the respective YIN and YANG pair. The following figure 11 illustrates this. The segment has two YIN-YANG pairs since the differences between Group1 and Group 4 is 50 while the differences between Group2 and Group 4 is 47.


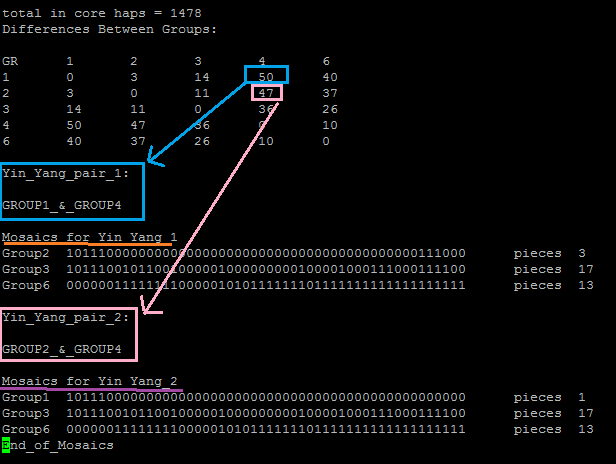


**Figure 11: screenshot showing portion of the output files named CORE_HAPS_New_x having >1 Mosaic**

In addition to the **Hap_Grouping_New_x** and **CORE_HAPS_New_x files,** other output files generated at the end of STEP II are **STAT_FOR_Mos_New_x** and **STAT_FOR_Yin_Yang_New_x, where x denotes the Chromosome number. These** output files contains all the statistics for Mosaic haplotypes and YIN-YANG haplotypes respectively. Another short script **combine_stats_YY_with_Mos.pl** is used to combine statistics for YIN-YANG and Mosaics together and generate the output file **Combined_STATS_YY_Mos_x**, x denoting the chromosome number.

22 different sets of 3 output files for each of the 22 autosomes are generated in this step and those files are again analyzed to calculate the statistical summaries in the **STEP IV**.

To view **STAT_FOR_Yin_Yang_New_1** see **Supplementary_File_S4.xlsx.**

To view **STAT_FOR_Mos_New_1** see **Supplementary_File_S5.xlsx.**

To view **Combined_STATS_YY_Mos_1** (for chromosome 1) see **Supplementary_File_S6.xlsx.**

STEP II requires 30 minutes to complete all computations and to generate outputs for chromosome 2. Thus, it takes less than 30 minutes for each of the remaining chromosomes.

**STEP III: Analyzing the Mosaic haplotypes and generating statistics for them**

This step uses a combination of 2 perl scripts to generate different statistics for the Mosaic haplotypes. One of those scripts is a system call which is used to execute the primary script for all relevant files. The script **system_for_ALL_MOSAIC.pl** is used to execute the primary script **Mosaic_STAT_generator_ALL_MOSAIC_GRs.pl** with the following command line:

**% perl system_for_ALL_MOSAIC.pl**

The primary script **Mosaic_STAT_generator_ALL_MOSAIC_GRs.pl** uses the **CORE_HAPS_New_x** files generated at the end of STEP II as the inputs and performs different statistical analysis of all the Mosaic haplotypes from all segments of a chromosome. The output generated in this step is **STAT_FOR_Mosaics_ALL_GROUPs_CHR_x** where x denotes the chromosome number. Following is a screenshot of one such output file


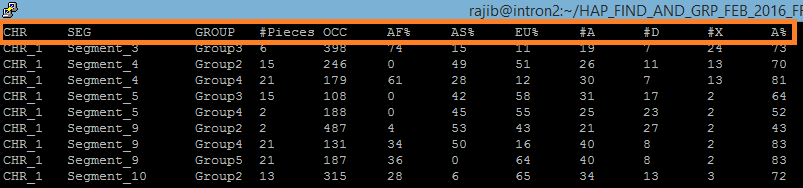


**Figure 12: screenshot of a prototype of the output files named STAT_FOR_Mosaics_ALL_GROUPs_CHR_x**

22 different output files for 22 chromosomes are generated in this step and those files are again analyzed to calculate the statistical summaries in the next step.

Similarly, two other system call-Perl script combinations are used to generate different statistics**.**

1. System call **system_for_MOST_ANCESTRAL_MOSAIC.pl** is used along with **Mosaic_STAT_generator_MOST_ANCESTRAL_MOSAIC.pl.** This also uses the **CORE_HAPS_New_x** files generated at the end of STEP II as the inputs and performs statistical analysis for the Mosaic haplotypes with most ancestral alleles in each segment. The output generated in this step is **STAT_FOR_Mosaics_MOST_ANCESTRAL_CHR_x** which has a similar structure as the **STAT_FOR_Mosaics_ALL_GROUPs_CHR_x** file shown above (with x being the chromosome number).
2. System call **system_for_TOP_PIECES_MOSAIC.pl** is used along with **Mosaic_STAT_generator_TOP_PIECES_MOSAIC.pl. T**his also uses the **CORE_HAPS_New_x** files generated at the end of STEP II as the inputs and performs different statistical analysis for the Mosaic haplotypes with maximum number of pieces in each segment. The output generated in this step is **STAT_FOR_mosaics_TOP_PIECES_CHR_x,** with x being the chromosome number. This output file also has a similar structure as the **STAT_FOR_Mosaics_ALL_GROUPs_CHR_x** file shown above.

22 different output files for 22 chromosomes are generated in both cases and those files are again analyzed to calculate the statistical summaries in the next step.

It takes 20 minutes to complete all STEP III computations and generate outputs for chromosome 2.

**STEP IV**: **Generating statistical summary for YIN, YANG and MOSAIC haplotypes.**

This step uses three different Perl scripts to generate summary statistics for Yin, Yang and Mosaic haplotypes.

**Yin_Yang_STAT_explorer.pl** uses **STAT_FOR_Yin_Yang_CHR_x** files (generated in STEP II) as inputs and computes the summary statistics for Yin and Yang haplotypes, which is saved in the output file **Analysis_YIN_YANG_Freq_0.25.**

This output file is transferred into the PC/Mac environment from the Linux environment in MS Excel format by **sftp** and further analyzed with MS Excel.

**Mosaic_STAT_explorer.pl** uses **STAT_FOR_Mosaics_ALL_GROUPs_CHR_x** files (generated in STEP III) as inputs and computes the summary statistics for all Mosaic haplotypes, which is saved in the output file **Analysis_Mosaics_All_Gr_Freq_0.25.** This output file is transferred into the PC/Mac environment from the Linux environment in MS Excel format by **sftp** and further analyzed with MS Excel.

Similarly, **Mosaic_STAT_explorer_2.pl** is used to generate summary statistics for Mosaic haplotypes with most ancestral alleles in each segment using the files **STAT_FOR_Mosaics_MOST_ANCESTRAL_CHR_x.**

**Mosaic_STAT_explorer_3.pl** is used to generate summary statistics for Mosaic haplotypes with maximum number of pieces in each segment using the files **STAT_FOR_mosaics_TOP_PIECES_CHR_x** as inputs.

The output files in these last two cases are named **Analysis_Mosaics_MOST_ANCESTRAL_Freq_0.25** and **Analysis_Mosaics_TOP_PIECES_Freq_0.25** respectively**.**

These output files are transferred into the PC/Mac environment from the Linux environment in MS Excel format by **sftp** and further analyzed with MS Excel.

All STEP IV computations are completed and all output files are generated within 25-30 minutes for chromosome 2.

**NOTE: For calculating different statistics for our study, we have used Mosaic_STAT_explorer.pl and Mosaic_STAT_explorer_3.pl.**

**STEP V: Building the Denisovan diplotypes and comparing them with the Human Common Haplotypes**

The vcf.gz files for the 22 Denisovan autosomes are stored in the following directory in our intron2 supercomputer workstation.


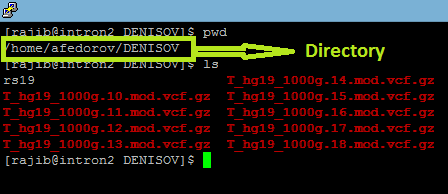


**Figure 13: screenshot showing Denisovan vcf.gz files in intron2 supercomputer workstation directory**

In this step, a Perl script **Deni_extract_SNP.pl** is used to extract all the SNPs and their relevant information from each of the 22 Denisovan autosomes. These were saved into 22 different output files named **DENISOVAN_Pinky_ALL_SNPs_x,** where x denotes the chromosome number.

The Perl script **DENISOVA_PINKY_HAPLO_FIND.pl** is used to find haplotypes for each of the 22 Denisovan autosomes and carry out further statistical analyses.

1. **Identifying human chromosomal segments which contain common haplotypes:**

The algorithm uses **STAT_FOR_Mosaics_ALL_GROUPs_CHR_x** files (generated in STEP III) to identify in a particular chromosome (x denotes the chromosome number) the segments where Yin, Yang and Mosaic haplotypes are present. Thus, the segments that do not have Mosaics are skipped since our goal here is to compare all the human common haplotypes with the Denisovan haplotypes. Note that the algorithm extracts only the segments that have one or more Mosaic haplotypes consisting of >=12 pieces. For example, let us take a look at the following figure 14. Segment 4 has 2 MOSAIC haplotypes with 15 and 21 pieces respectively, while segment 5 has 1 MOSAIC haplotype with 15 pieces. The algorithm selects these segments but skips segments 3, 5 and 9 since the MOSAIC haplotypes in those segments consist of <12 pieces.

**
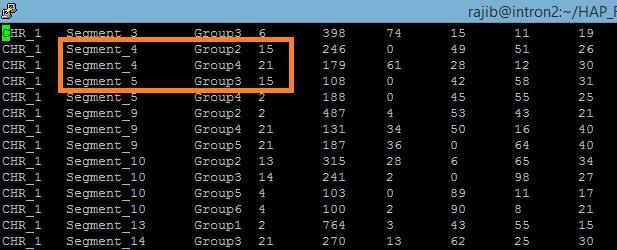
**

**Figure 14: identifying human chromosomal segments having CHs**

1. **Selecting human haplotype SNPs**

Next, as a second input file, the algorithm opens the **ALL_STATS_Cx-y** files for the respective segments identified above **(**where x is the chromosome number and y is the segment number). Thus, for the scenario shown in the picture above, the program opens the **ALL_STATS_C1-4** file (see figure 15 below). From each **ALL_STATS_Cx-y** file, the program extracts the 50 SNP ids [denoted as rs12345 for known SNP id or dot (.) for unknown SNP id].


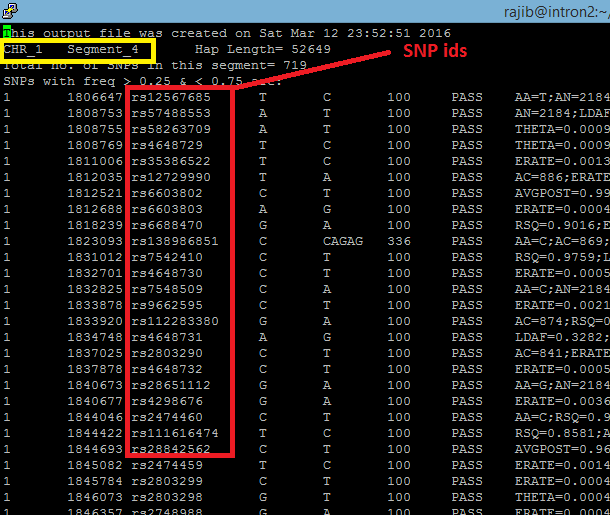


**Figure 15: Extracting SNP ids**

1. **Constructing the Denisovan diplotypes:**

The program then opens the third input file **DENISOVAN_Pinky_ALL_SNPs_x**, where x is the chromosome number. In the current example, the program will open the **DENISOVAN_Pinky_ALL_SNPs_1** file. The algorithm scans this third input and searches for the extracted SNPs with a known SNP id (identified above). When a match is found in the Denisovan genome, the algorithm extracts the genotype information for the respective SNP and saves it. Since the Denisovan genome sequence data is unphased, the algorithm translates the genotype information into a diplotype instead of haplotype. Genotype annotations corresponding to those GVs with respect to homozygous with genetic variants (annotated 1/1 in the VCF file) are translated into 2 (1+1), homozygous presence of reference alleles (annotated 0/0) are translated into 0 (0+0) and heterozygous (annotated 0/1 or 1/0) are translated into 1 (0+1 or 1+0). SNP for which no match is found is noted; and an ‘x’ is saved for its position in the diplotype.

The Denisova genome contains some SNP ids in duplicate. To avoid complications, we decided not to include those SNPs in our analysis. Therefore, a ‘U’ is saved in the diplotype for such SNPs.

The Denisova genome also contains incomplete genotype information for some SNPs (1/. Or ./1 or 0/. Or ./0). We also excluded those SNPs from our analysis. The algorithm saves an ‘N’ in the diplotype for each SNP with incomplete genotype information in the Denisova genome. Figure 16 below exemplifies such an instant.


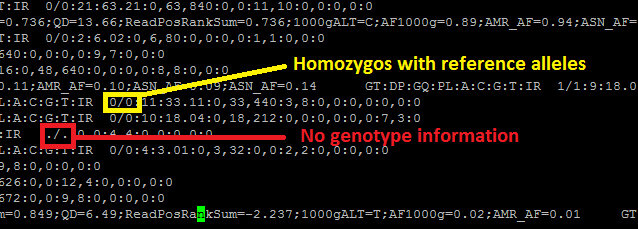


**Figure 16: screenshot showing missing genotype information in one of the Denisovan chromosome data files**

For SNPs which has unknown SNP id (dots) in the human genome are represented in the diplotype with an ‘x’. The SNP positions for which no match is found in the Denisovan genome are also denoted as ‘x’.

The algorithm also computes the ancestral and derived state of each allele in the Denisovan diplotype by processing the ancestral allele information from the **CORE_HAPS_New_x** file for the respective segment.

The Denisovan diplotype and the computed ancestral state is then printed along with the contents of the **CORE_HAPS_New_x** file into the output files named **CORE_HAPS_with_DENI_Pinky_x,** where x is the segment number. Figure 17 shows a portion of one such output file.


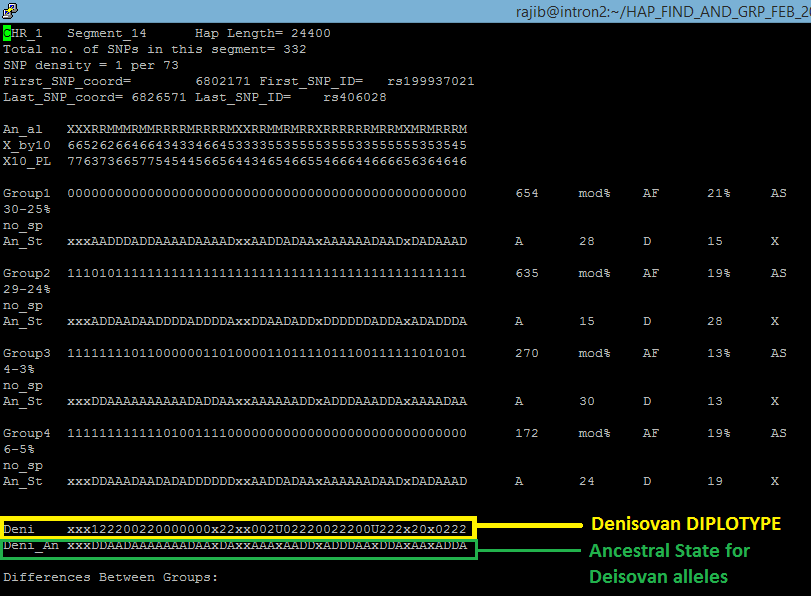


**Figure 17: screenshot showing a prototype of output files named CORE_HAPS_with_DENI_Pinky_x**

1. **Generating comparative statistics for denisovan:**

A system call **system_for_DENI_compar_STAT_gen.pl** is used to execute the third Perl script in STEP V, namely **Deni_STAT_generator.pl**. This processes all the **CORE_HAPS_with_DENI_Pinky_x files** for each chromosome and generates the statistics. The command line used is as follows

**%perl system_for_DENI_compar_STAT_gen.pl**

The results are saved in an output file named **STAT_Comparison_FOR_DENI_CHR_x** (x denotes the respective chromosome number). Following figure 18 is a screenshot of one such output file:


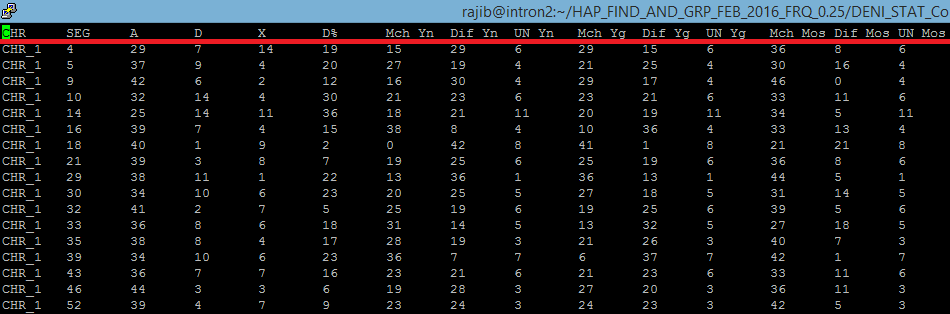


**Figure 18: screenshot showing a prototype of the output files named STAT_Comparison_FOR_DENI_CHR_x**

Finally, the Perl script **DENI_STAT_explorer.pl** processes 22 such **STAT_Comparison_FOR_DENI_CHR_x** files (one for each chromosome) to compute the summary statistics and saves into the output file named **Analysis_DENI_All_Freq_0.25.** These output file is transferred into the PC/Mac environment from the Linux environment in MS Excel format by **sftp** and further analyzed with MS Excel.

STEP V requires 90 minutes to complete all computations and generate outputs.

**STEP VI: Identifying the presence of Ancestral haplotypes in the Human population**

Ancestral allele information is denoted by the nucleotides A, C, G and T (see green boxes in the figure 19 below) in the 1000Genomes .vcf.gz files. For some SNPs, the ancestral state is not known. The 1000Genomes database denotes ancestral information for such SNPs by a dot [.] (see yellow boxes in the figure 19 below).


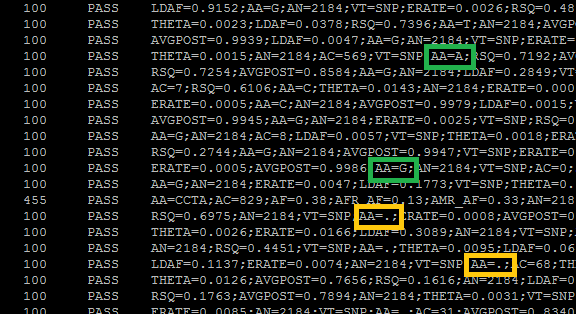


**Figure 19: screenshot showing missing ancestral allele information in one of the 1000Genome .vcf.gz file**

Ancestral allele information for each SNP of interest is extracted in STEP I by the Perl script ***Haplo_find.pl*** which further processes this information and computes an ancestral haplotype for each segment. This ancestral haplotype is a string of 50 SNPs that represent only the ancestral alleles for that particular segment. This ancestral haplotype is saved in the STEP I output files named **ALL_STATS_Cx-y** (where x denotes Chromosome number and y denotes the segment number). The *Perl* program ***Ancestral_Hap_Match_Finder.pl*** extracts the ancestral haplotype for each segment from the respective **ALL_STATS_Cx-y** file and finds its matches from the 2184 haplotypes in the 1000Genomes population in the corresponding segment. The algorithm compares only the alleles for which the ancestral state is known. It does not consider the alleles with unknown ancestral state. For example, if the ancestral haplotype of a particular segment contains 15 or more alleles with unknown ancestral state, this program skips that segment. Also, this program allows <=2 mismatches while searching for matches. For example, let us consider a segment that has an ancestral haplotype consisting of 40 alleles with known ancestral state and 10 alleles with unknown ancestral state. In this case, the algorithm looks for matches of the ancestral haplotype for those 40 positions. The haplotype that matches 38, 39 or 40 of those alleles is considered a ‘match’.

The primary script **Ancestral_Hap_Match_Finder.pl** is executed for all segments in a chromosome with the help of the system call **system_for_Anc_Hap_Match_finder.pl.** The system call provides the user with the option to select the desired chromosome and total number of segments intended to be considered. The Command line for execution of this script is

**%perl** **system_for_Anc_Hap_Match_finder.pl. 1 456**

Here the first argument (1) represents the chromosome number and the second argument (456) represents the total number of segments for chromosome 1 (generated in STEP I).

The first output file generated is **MATCH_Anc_Hap_CHR_x_2mm** (x denotes the chromosome number). For example, see figure 20 below.

**
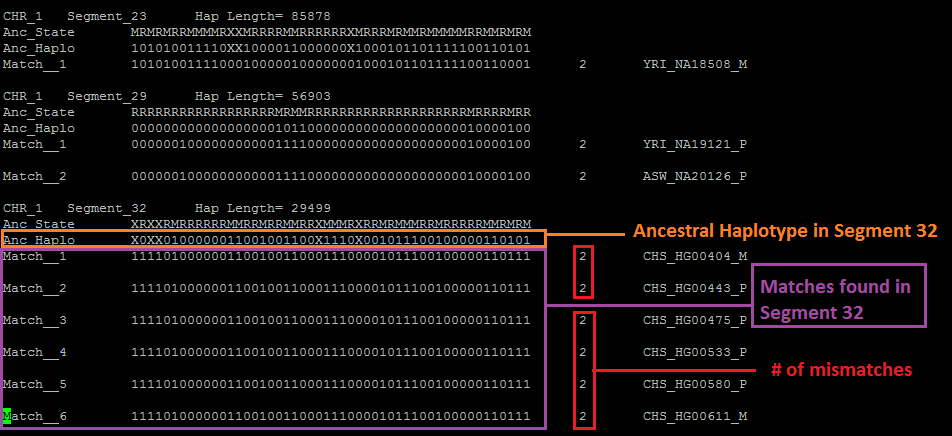
**

**Figure 20: screenshot showing a portion of the output file MATCH_Anc_Hap_CHR_1_2mm**

Another output file **No_Match_Anc_Hap_CHR_x_2mm** (x denotes the chromosome number) lists the segments where no ancestral haplotype match was found.

A third output file **CONTINENT_STATs_Anc_Hap_All_CHR_2mm** is also generated which stores the continental distribution of ancestral haplotype matches for all segments from all the 22 chromosomes. Figure 21 below shows a part of this output file:


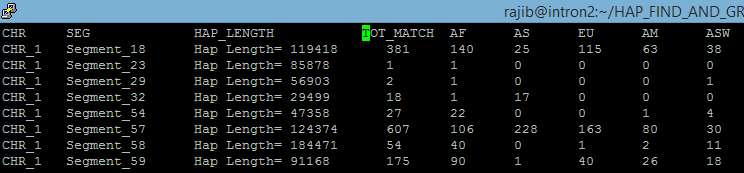


**Figure 20: screenshot showing a portion of the output file CONTINENT_STATs_Anc_Hap_All_CHR_2mm**

**Note:** Since phasing can be error prone sometimes, we identified the individuals who are homozygous for all the 50 SNPs in a segment and computed the diplotypes of these homozygous people. The homozygous diplotype is practically the sum of two identical haplotypes and therefore, is not susceptible to phasing error. We compared these diplotypes with the haplotypes in each segment to have a control for phasing errors. We found the phasing in the haplotypes to be consistent with the diplotypes in >99% of cases. These homozygous diplotypes are listed at the end of each **ALL_STATS_Cx-y** output file generated in STEP I.

**List of all Perl scripts used in this study:**

***HaploFind.pl;***

***HapGroupGenerator.pl,***

***MosaicStatGenerator1.pl,***

***MosaicStatGenerator2.pl,***

***MosaicStatGenerator3.pl,***

***YinYangStatExplorer.pl,***

***MosaicStatExplorer.pl,***

***MosaicStatExplorer_2.pl,***

***MosaicStatExplorer_3.pl,***

***CombineStatsYY_Mos.pl,***

***AncestralHapMatchFinder.pl*.**

***Deni_extract_SNP.pl***

***DENISOVA_PINKY_HAPLO_FIND.pl***

***Deni_STAT_generator.pl***

***Deni_STAT_explorer.pl***

***PROTOCOLS for GEMA Simulations***

For GEMA simulations we used MAGEvFinal_01.pl program using the command line:

% nohup perl MAGEvFinal_01.pl InputParameters.txt &

Such programs were running for 2-50 days depending on the parameters that are specified in the file InputParemeters.txt:

%more InputParameters.txt

1 SelCoeff OutPutD PopuSize Offspr NumMut NumRecom DomiCoeff GeneSize

A ExpC_6MB.txt 124-5-20-48-0.5-10000-ExpC_6MB 124 5 20 48 0.5 10000

This program automatically creates output directory (OutPutD), which is in this example has the name 124-5-20-48-0.5-10000-ExpC_6MB.

In addition, it takes the input file for the distribution of selection coefficients (ExpC_6MB.txt), which is 126MB and located in the INTRON2 workstation (dir: /home/afedorov/GEMA2016). This ExpC_6MB.txt file was used for all modeling experiments. The GEMA parameters are specified in the InputParameters.txt file. Specifically, in this example ***N***=124; ***α***=5; ***μ***=20; ***r***=48; ***h***=05; and gene length is ***L***=10,000 nts.

Upon its execution the program outputs and periodically updates three files: result_OUT, backup_A, and backup_B.

%tail result_OUT

Generation Fitness #SNPs #total fix mutations

1160 124011577.5 807079 17115

1170 125242531.5 802091 17549

1180 126472138 804990 17915

1190 127696912.5 802986 18314

1200 128926400.5 806629 18751

1210 130152164.5 802784 19131

1220 131388909.5 804263 19517

1230 132628784.5 805956 19915

1240 133863215 800997 20291

1250 135108877 802750 20695

The program has been stopped when 1) the total number of SNPs (third column in the result_OUT) is on a plateau; and 2) number of the fixed mutation per generation (see fourth column) reached ***k=2μ***. In this example it is (20695-20291)/10. When the selection is absent (***α*** =2) the second requirement is reached very slowly. So, even for ***N***=250, ***α***=2, we waited nearly 4,000 generations (about ten days).

At the end of simulation experiment we used backup_A file to calculate frequent SNPs and their haplotypes. With this aim four Perl scripts has been executed consequently:

1)

% perl GemaBackupA_Process.pl backup_A >GEMA124-5-20-48-0.5-10000-ExpC_6MB

It creates output file GEMA124-5-20-48-0.5-10000-ExpC_6MB, used as input in the next step:

2)

% perl YinYangGEMA.pl GEMA124-5-20-48-0.5-10000-ExpC_6MB 124-5-20-48-0.5-10000-ExpC_6MB 124

Here input file is the first argument ($ARGV[0]), the second argument ($ARGV[1]) is the part of the name of the output file to which “GEMA_HAPLOTYPES” string will be “ligated”. Finally, the third argument ($ARGV[2]) is the size of the population (in this example 124). The output file for this step is GEMA_HAPLOTYPES_124-5-20-48-0.5-10000-ExpC_6MB

3)

% perl GemaSegments.pl GEMA_HAPLOTYPES_124-5-20-48-0.5-10000-ExpC_6MB

It takes as input the output from the previous program GEMA_HAPLOTYPES_124-5-20-48-0.5-10000-ExpC_6MB (which is the argument in the command line) and creates a series of output files which number is equal to the number of segments.

The output files look like:

seg_GEMA_HAPLOTYPES_124-5-20-48-0.5-10000-ExpC_6MB_1

seg_GEMA_HAPLOTYPES_124-5-20-48-0.5-10000-ExpC_6MB_2

…

seg_GEMA_HAPLOTYPES_124-5-20-48-0.5-10000-ExpC_6MB_204

where the total number of segments is 204.

4)

% perl GEMAhaplotypes.pl GEMA_HAPLOTYPES_124-5-20-48-0.5-10000-ExpC_6MB Aug5

Here the first argument ($ARGV[0]) shows the name of the series of files to be open. This string will be automatically appended to the string “seg_” and all the files in this series will be opened and processed. The second argument ($ARGV[1]) is the prefix to the series of output files to which the string of ($ARGV[0]) will be appended. These output files look like:

Aug5_GEMA_HAPLOTYPES_124-5-20-48-0.5-10000-ExpC_6MB_1

Aug5_GEMA_HAPLOTYPES_124-5-20-48-0.5-10000-ExpC_6MB_2

…

Aug5_GEMA_HAPLOTYPES_124-5-20-48-0.5-10000-ExpC_6MB_204

where the total number of segments is 204.

These output files contain the haplotypes of all simulated virtual individuals ranked by their occurrence. For example:

% more July25GEMA_HAPLOTYPES_2000-5-20-48-0.5-10000-ExpC_6MB_591

SEGMENT 591 haplotypes

101111011111111111111000111101 113

110000100000111111111000111101 62

110111111100000000000111001100 47

110111111111100100010000100000 39

011110111111111111111000111101 39

110000110000111111111000111101 39

111000110000111101011000100010 37

011000100000111111111000111101 32

111111000000111111111000111100 31

….

…

000000101111100100011001000011 1

011000101111000000000111000001 1

001111111111000000000111001100 1

110100011111111111111000100000 1

011111111111111100111111000100 1

**NOTES:** I) This Perl pipeline calculates frequent SNPs (MAF >25%) and put them into the haplotypes of 30 characters (see picture above). In the simulation experiments we used 30 neighboring SNPs instead of 50 SNPs as in 1000 Genomes individuals because the occurrence of CHs are much rarer in simulations. (Using 50 SNPs often results in no CHs and Yin/Yang pairs). We also lower the threshold for the grouping of similar haplotypes in the next two Perl programs (*GemaHapGroupGenerator.pl, Gema_HapGrouping.pl*), where we considered two haplotypes with 1-4 differences belonging to the same group.

II) Since the size of modeling populations are limited (N<= 2000) the total number of SNPs and frequent SNPs are not large enough compared to humans. Therefore, to get statistics we did not use segments, yet instead haplotypes are followed each other without any space.

5)

A system call system_for_GEMA_HAP_GROUPING.pl is used here to execute the primary Perl script named GEMA_Hap_Group_generator.pl. This script uses the output files generated in the previous step and computes the haplotype groups, determines the common haplotypes (Yin, Yang and Mosaic) and calculates different summary statistics.

The command line used is:

**%perl system_for_GEMA_HAP_GROUPING.pl**

The results are saved in the output files:

**vi STAT_for_Y_Y_GEMA_HAPS_JUNE_10**

Seg #cores Tot_Occ_cores max_diff #Yin_Yang #Mosaics

3 10 707 28 3 8

16 10 763 27 1 8

21 9 748 28 2 7

25 7 870 28 1 5

88 6 372 27 1 4

91 3 332 29 1 1
