# Supplementary material for: Intricacies in arrangement of SNP haplotypes suggest “Great Admixture” that created modern humans
Source: BMC Genomics. 2017 Jun 5;18:433. doi: 10.1186/s12864-017-3776-5 (PMC5741169; doi:10.1186/s12864-017-3776-5)

**SUPPLEMENTARY FILE SD2**

***PROTOCOL for Reconstruction of Ancestral Lineages using Machine Learning***

In an attempt to reconstruct these ancestral lineages, we used the Machine Learning approaches such as K-means Clustering and Decision Tree Classifiers to characterize the clusters that may correspond to these hypothetical lineages. Weka and Rapid Miner programs were used for this purpose.

Five normalized parameters for Yin and Yang haplotypes for each segment (total haplotype occurrence; the number of derived alleles; percentage of haplotype occurrence in Africa, Asia, and Europe) have been studied. These results and details are provided in the below.

**Figure 1. Clustering of Normalized Parameters for Yin and Yang Haplotypes.***The following table shows the normalized parameters that were used for K-means Clustering. The first column has normalized values for haplotype occurrence, the second through fourth columns contain normalized values for continent occurrence, the fifth column contains the derived percentage, and the last column contains the cluster that each row was classified into.*


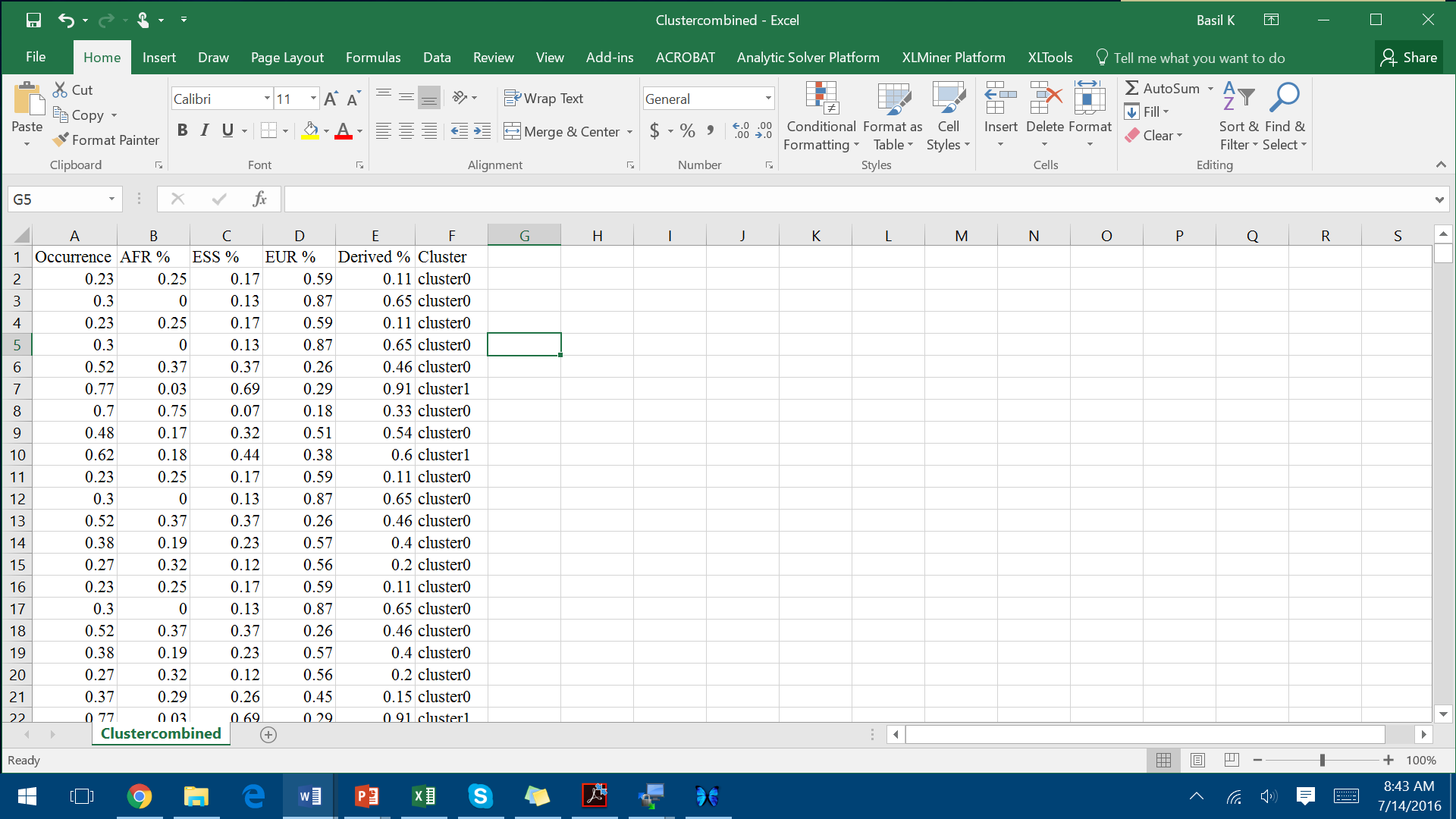


**Figure 2. Visualization of Clusters created through K-means Clustering in Weka.***Figure 2 showcases visualization of the clusters generated by the Weka software. The X and Y axises contain the individual classifiers plotted against one another.*


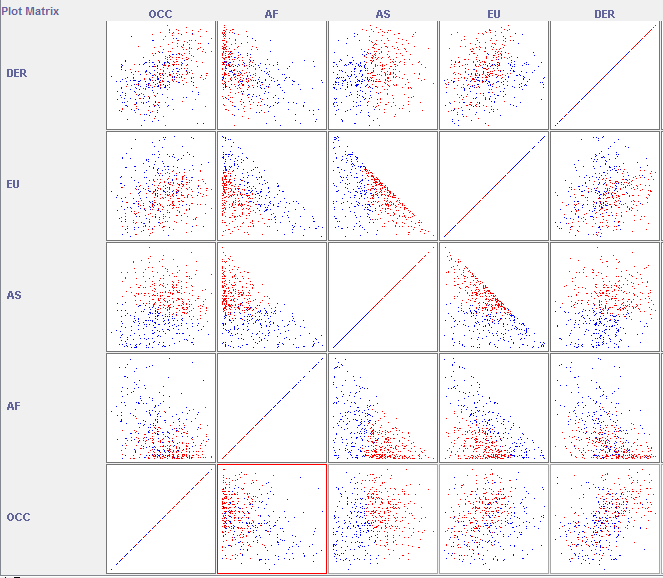


**Figure 3. Decision Tree created by Rapid Miner.***The following image shows the Decision Tree that was created through the Rapid Miner program. Decision Tree Classification is a form of Supervised Machine Learning. To create the tree, the clustering that was created using K-Means Clustering, as seen in Figure 1, was used as the input variable.*


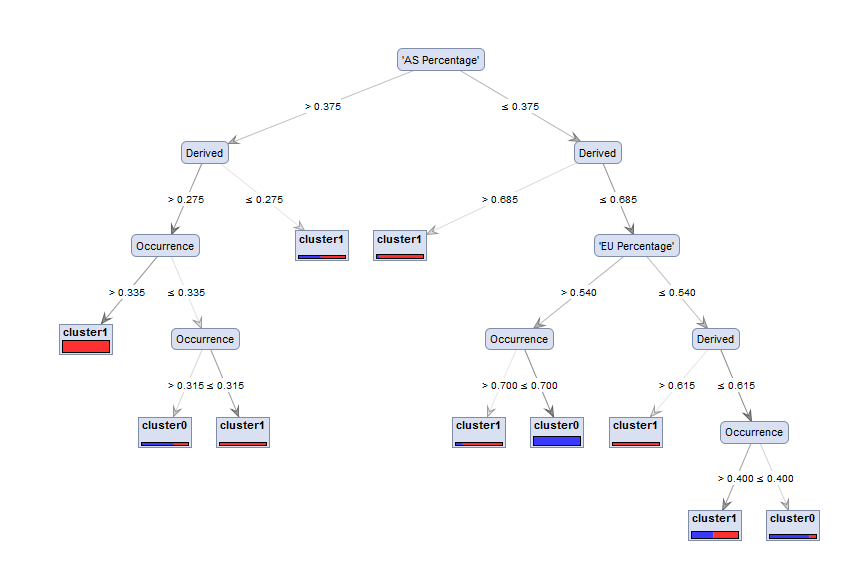

Supplement: Supplementary file 5 — The results of Machine Learning approaches (Weka, (26) and Rapid Miner, (27) web computational resources) are presented here. (DOCX 238 kb) [file 12864_2017_3776_MOESM5_ESM.docx]
